# Probing quantum mechanics with nanoparticle matter-wave interferometry

**DOI:** 10.1038/s41586-025-09917-9

**Published:** 2026-01-21

**Authors:** Sebastian Pedalino, Bruno E. Ramírez-Galindo, Richard Ferstl, Klaus Hornberger, Markus Arndt, Stefan Gerlich

**Affiliations:** 1 Faculty of Physics, University of Vienna, Vienna, Austria; 2 Vienna Doctoral School in Physics, University of Vienna, Vienna, Austria; 3 Faculty of Physics, University of Duisburg-Essen, Duisburg, Germany

**Keywords:** Matter waves and particle beams, Quantum mechanics, Nanoparticles

## Abstract

The quantum superposition principle is a fundamental concept of physics^1^ and the basis of numerous quantum technologies^2,3^. Yet, it is still often regarded counterintuitive because we do not observe its key features on the macroscopic scales of our daily lives. It is, therefore, interesting to ask how quantum properties persist or change as we increase the size and complexity of objects^4^. A model test for this question can be realized by matter-wave interferometry, in which the motion of individual massive particles becomes delocalized and needs to be described by a wave function that spans regions far larger than the particle itself^5^. Over the years, this has been explored with a series of objects of increasing mass and complexity^6–9^ and a growing community aims at pushing this to ever larger limits. Here we present an experimental platform that extends matter-wave interference to large metal clusters, a qualitatively new material class for quantum experiments. We specifically demonstrate quantum interference of sodium nanoparticles, which can each contain more than 7,000 atoms at masses greater than 170,000 Da. They propagate in a Schrödinger cat state with a macroscopicity^10^ of *μ* = 15.5, surpassing previous experiments^5,9,11^ by an order of magnitude.

## Abstract (web summary)

Quantum interference of sodium nanoparticles, which can each contain more than 7,000 atoms at masses greater than 170,000 Da, is demonstrated.

## Main

When Louis de Broglie postulated that we need to ‘associate a periodic phenomenon with any isolated portion of matter or energy’, he predicted that these new ideas would ‘solve almost all the problems brought up by quanta’^12^. The quantum wave function has become a core concept in modern physics^13^ and has withstood all tests to date. However, it is still a matter of debate whether quantum physics is already the ultimate theory or if it needs to be extended to explain its transition into classical phenomena. This debate has sparked general interest in the scientific community, shown by a series of recent experiments that have pushed the limits of quantum mechanics. Single atoms were delocalized on the half-metre scale^5^ or for times longer than a minute^14^. Matter-wave interference was seen in complex molecules, from fullerene diffraction^6^, to interference with biomolecules^15^, van der Waals clusters of organic molecules^8^ and families of fluorinated oligoporphyrins^9^. Mechanical cantilevers were cooled to their quantum ground state, both cryogenically^16^ and optically^17^. Crystal oscillators^18^ and levitated nanoparticles were cooled to the lowest level of their harmonic motion in one or two degrees of freedom^19–23^. Recently, the vibration mode of a bulk acoustic resonator was prepared in a quantum superposition state, with an effective mass of 16 μg (10^19^ Da) (ref. ^24^) delocalized over 10^−18^ m.

Here, we present our work on nanoparticle interferometry in a complementary regime. In our case, the centre-of-mass position of clusters containing more than 7,000 atoms becomes delocalized over a distance exceeding the diameter of the particle by more than an order of magnitude. This quantum state is analogous to Schrödinger’s cat: here, a macroscopic object that defies intuition because it involves a superposition of classically distinct trajectories^25^.

The unique combination of mass and delocalization is particularly well suited for probing theories that modify the Schrödinger equation through nonlinear and stochastic terms to suppress macroscopic superpositions^4^. These macrorealistic models have been proposed as a solution to the quantum measurement problem^26^ as they would explain why very massive objects are always found in a well-defined position^27^. They assume that the wave function collapses to a localized state, spontaneously^28^ or induced by gravity^29,30^, such that a nanoparticle in an interferometer would lose its quantum coherence and the interference fringes would fade. The larger the mass or the longer the propagation time, the stronger the predicted loss of visibility. In our present experiment, we observe interference of widely delocalized massive particles, demonstrating that standard quantum mechanics holds at this scale with no need to modify the Schrödinger equation. We quantify the size of our superposition in terms of quantum macroscopicity, a measure that provides a unified framework for constraining a wide range of macrorealist modifications. The observed macroscopicity exceeds that of all previous quantum experiments by an order of magnitude^31^.

## Experiment

**Fig. 1:**
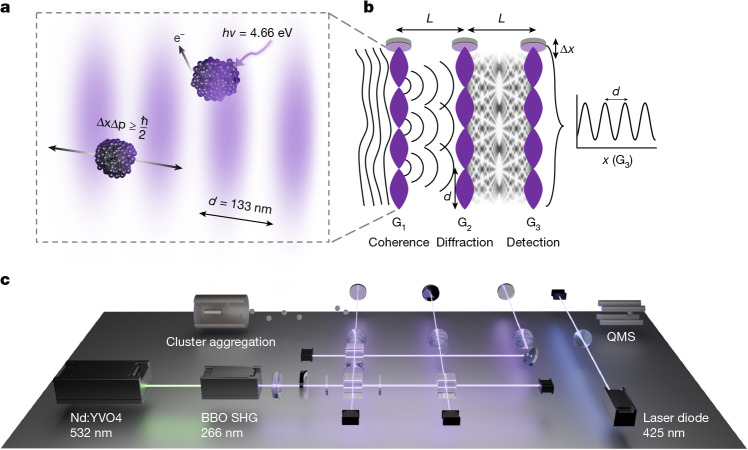
Experimental overview. **a**, Photo-ionizing gratings as beam splitters. Clusters passing through the antinodes of the optical grating are ionized and removed, whereas those passing through the nodes remain neutral. This confines particles to a spatial region within the grating nodes, resulting in a momentum uncertainty. The light field also induces a dipole moment, imprinting a position-dependent phase onto the clusters. **b**, Schematic of an optical Talbot–Lau interferometer. Starting with incoherent matter waves, the first grating (G_1_) prepares coherence by spatially confining the particles, as described in **a**. Transverse coherence grows towards G_2_, behind which a Talbot–Lau carpet emerges in the near field. Finally, a third grating acts as a position-resolving detection mask scanned across the interference pattern. **c**, Schematic of the multiscale cluster interference setup. An effusive sodium source in an aggregation chamber generates the cluster beam. The beam is transmitted through several differential pumping stages into the interferometer chamber kept at ultrahigh vacuum conditions (about 9 × 10^−9^ mbar). The cluster beam overlaps with three perpendicular standing light waves equally spaced at a distance of *L* = 0.983 m, forming optical gratings with a period of *d* = 133 nm. The intensities of the first and third gratings are chosen such that they act as absorptive gratings, whereas the second grating is operated at lower laser intensity, realizing an optical phase grating. After passing through the interferometer, the remaining neutral clusters are photo-ionized using a 425 nm laser diode and mass-filtered. The third grating is scanned transversely across the molecular beam. The integrated signal is then recorded as a function of the displacement of the grating. The nanoparticle and optical components in **a** and **c** were rendered in Blender using assets by Ryo Mizuta Graphics.

The de Broglie wavelength *λ*_dB_ = *h*/*mv* of a matter-wave beam is determined by Planck’s constant *h*, the particle mass *m* and its velocity *v*. Matter-wave interference at high masses requires both the preparation of low particle velocities and the ability to handle short de Broglie wavelengths. In our multiscale cluster interference experiment (MUSCLE), we achieve this by combining a cryogenic metal cluster source with three ultraviolet (UV) diffraction gratings in a Talbot–Lau configuration, shown in Fig. 1.

Cluster aggregation sources enable scalable synthesis of particles across a wide mass range, and they are versatile in handling a variety of materials^32,33^. Here, we prepare sodium clusters consisting of 5,000–10,000 atoms, in a helium–argon mixture at 77 K. They travel at velocities around 160 m s^−1^ with de Broglie wavelengths between 10 fm and 22 fm.

The short de Broglie wavelength makes far-field diffraction challenging even for grating periods on the 100 nm scale: it would require beam collimation to below 200 nrad. However, in 1997, John Clauser proposed using near-field interferometry for grating-based coherent self-imaging of ‘small rocks and live viruses’^34^, noting that this approach is compact, tolerates initially incoherent beams and offers high spatial resolution. This has been demonstrated with atoms^35,36^, X-rays^37^, positrons^38^, as well as organic and tailored macromolecules^7,9^. Here, we use it to open a window to matter-wave research with a whole new class of quantum objects, namely, massive metal nanoparticles.

A Talbot–Lau interferometer is built from three gratings with period *d* and spacing close to the Talbot distance *L*_T_ = *d*^2^/*λ*_dB_ (ref. ^39^). The first and third gratings act as periodic spatial filters to prepare matter-wave coherence in G_1_ and to resolve the interference fringes that emerge at G_3_. The second grating G_2_ modulates the amplitude and phase of the cluster matter wave. Standing light waves are favoured over nanomechanical diffraction gratings because their period is precisely defined, and their transmission amplitude can be modified in situ.

In contrast to atom interferometry, where optical beam splitters are commonly tailored to specific electronic transitions^40,41^, ionization and phase gratings are compatible with a large variety of materials and particle sizes. Ultraviolet light serves well as an amplitude or photodepletion grating when the clusters in the antinodes are ionized and discarded. The standing light field additionally induces an oscillating dipole moment in the transmitted clusters, in proportion to their optical polarizability. Thus, it also imprints a spatially periodic phase shift onto the de Broglie wave associated with each nanoparticle.

The light for the three gratings is derived from a single-line green laser beam, which is frequency doubled in an external cavity to produce up to 1 W of power at 266 nm. It is split into three partial beams, which are retro-reflected to form three standing light waves, separated by 0.983 m. Neutral clusters transmitted by the interferometer are photo-ionized and counted by a quadrupole mass spectrometer using a conversion dynode and electron multiplier.

We sample the interference patterns by scanning G_3_ across the cluster beam while counting the number of transmitted clusters as a function of the G_3_ position. The resulting fringes are phase stable to within 3–5 nm over several hours and can be fitted with a sinusoid to determine the visibility $V=({S}_{\max }-{S}_{\min })/({S}_{\max }+{S}_{\min })$, where *S*_max_ and *S*_min_ are the maximum and minimum of the fit, respectively.

### Interference scans

**Fig. 2:**
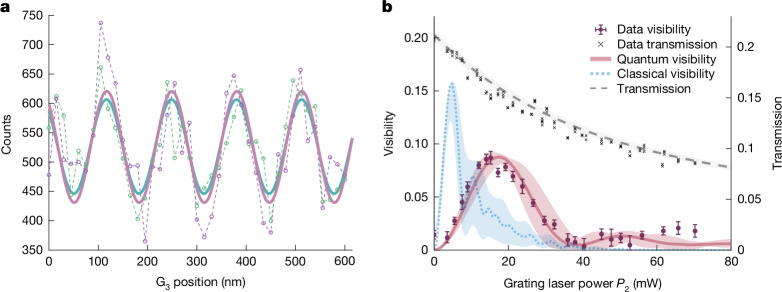
Interference results. **a**, Interference fringes of sodium clusters with a mean mass of 172 kDa. The experimental data of two independent measurement runs (purple and green dots) are fitted by a sine function (purple and green line) with a visibility of *V* = 0.10 ± 0.01 and *V* = 0.08 ± 0.01, for grating laser powers *P*_1_ = (62 ± 2) mW, *P*_2_ = (15.2 ± 0.3) mW and *P*_3_ = (68 ± 2) mW. **b**, Fringe visibility versus grating laser power of G_2_. Each data point shows the weighted mean visibility per power bin from multiple independent interference scans of sodium clusters with masses centred around 172 kDa. Visibilities and error bars are derived from per-measurement 1*σ* confidence intervals of nonlinear least square sine fits (Methods). G_1,3_ powers as above. The continuous red and the dashed blue lines show the expected interference contrast according to the quantum and the classical model, respectively. The shaded areas show the uncertainties of the theory curve, based on the experimental 1*σ* limits of the molecular velocity, mass distribution, absorption cross-section and optical polarizability. In this plot, both theory curves were scaled by the same global factor of 0.78.

In Fig. 2a, we show two representative interference fringes of sodium clusters with a diameter around 8 nm and masses ranging from 143 kDa to 197 kDa. We have measured a fringe visibility of up to *V* = 0.10 ± 0.01, which is limited by the finite photodepletion efficiency in the first and third gratings.

The observation of fringes in the cluster density distribution alone does not provide sufficient evidence for wave-like quantum propagation. They could also be explained by models in which the particles follow classical trajectories. In the presence of three nanomechanical gratings, classical flight paths would produce moiré-like shadow patterns. A similar classical picture is conceivable for sinusoidal transmission gratings in G_1_ and G_3_ and a phase grating in G_2_, in which the latter acts as an array of microlenses because of the optical dipole force.

To obtain clear evidence for the wave nature of the observed fringes, their visibility is analysed as a function of the laser power *P*_2_ of the second grating, shown by the solid circles in Fig. 2b. We compare this to the contrast predicted by both the classical (blue dotted line) and the quantum model (solid red line). The quantum model is obtained by describing the matter-wave dynamics in phase space using the Wigner–Weyl formalism^42^. It accounts for all coherent and incoherent grating interactions and enables a direct comparison with the prediction of classical mechanics (Methods).

We account for the experimental constraints on velocity, ionization cross-section, mass distribution and polarizability by the shaded areas along the theory curves. Interferometer misalignment, gravitational and rotational phase averaging, mechanical vibrations and the scattering of gas particles and thermal radiation can reduce the predicted contrast (Supplementary Information). We take this into account by a global scale factor of 0.78, which is equally applied to the quantum and the classical prediction in this figure. With this single experimental factor included, our experiments are well described by the quantum model and clearly distinct from the classical prediction.

Our assumptions regarding the mass, size and velocity distributions of the clusters, as well as the mass dependence of their ionization cross-section, are independently supported by the measured transmission probability as a function of the laser power in G_2_ (dashed black curve). The model reproduces the experimental data (black crosses) very well, without any additional scaling factor.

For substantially more massive clusters, with masses between 400 kDa and 1 MDa, we observe even higher fringe visibilities of *V* = 0.66 ± 0.09 (Supplementary Information). Although this may seem counterintuitive, it becomes plausible when we consider that the ionization cross-section increases and the transmissive regions in each grating become narrower with increasing size of the cluster.

**Fig. 3:**
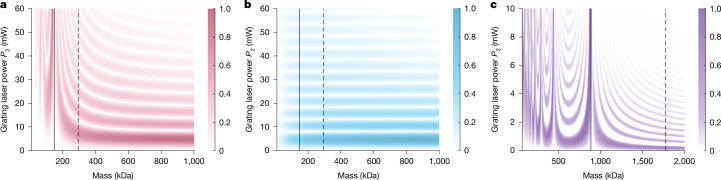
Predicted fringe visibility as a function of cluster mass and G_2_ laser power. **a**,**b**, Results are shown for the quantum model (**a**) and the classical model (**b**), which both include the effects of ionization and of the dipole force in the grating interaction. Both calculations assume a mean velocity of 160 m s^−1^, a Gaussian velocity spread of 10 m s^−1^ and grating powers of *P*_1_ = *P*_3_ = 100 mW. The solid line marks the mass at which the Talbot length equals the interferometer length, whereas the dashed line indicates the mass for which half the Talbot length coincides with the interferometer length. The colour scale indicates fringe visibility *V*. For masses beyond the Talbot condition, the quantum and classical models converge. **c**, Slowing the particles to approximately 25 m s^−1^ will enable our setup to reliably distinguish quantum from classical dynamics for masses exceeding 1 MDa.

However, the de Broglie wavelength in this mass range (*λ*_dB_ ≱ 3 fm) is too short to distinguish quantum from classical predictions, for our interferometer configuration (Fig. 3). For *L* ≤ *L*_T_, near-field matter-wave dynamics gradually transitions to geometrical optics, in agreement with Bohr’s correspondence principle^43^.

Figure 3 shows how the predicted visibilities from quantum (Fig. 3a) and classical theory (Fig. 3b) converge at high cluster masses. At the same time, it highlights a clear discrepancy between quantum and classical predictions in the mass range below 200 kDa (Fig. 2b). In Fig. 3c, we show that it will become possible to unambiguously demonstrate the quantum wave nature of clusters in the MDa range if their velocities can be reduced to about 25 m s^−1^.

## Discussion

While Schrödinger speculated about the possibility of a cat being ‘dead and alive’ in the same quantum state—something clearly impossible to observe in our macroscopic world—early experiments with trapped ions^44^ and cavity fields^45^ already showed that such superpositions can exist in microscopic systems. Here, we took this idea to a much more massive scale: a nanometer-sized piece of metal being ‘here and there’ in the same quantum state with a 133 nm separation between the two locations, more than an order of magnitude greater than the particle itself. What would seem impossible in a classical worldview becomes here an experimental fact of quantum physics.

Observing matter-wave interference of the most massive objects to date reveals no breakdown of the quantum superposition principle related to mass or size alone. Moreover, this work establishes a new platform for metal nanoparticles, a material class previously inaccessible to such tests, and it suggests the feasibility of quantum-interference experiments with complex nanobiological objects which cover a similar mass range.

To put our experiment into context with other demonstrations of quantum superposition states, we evaluate the macroscopicity measure *μ* as defined in refs. ^10,31^. This value quantifies to what extent a given quantum experiment probes the validity of quantum mechanics and how well it can exclude minimal modifications of the Schrödinger equation, which would break the quantum superposition principle at some macroscopic scale.

**Fig. 4:**
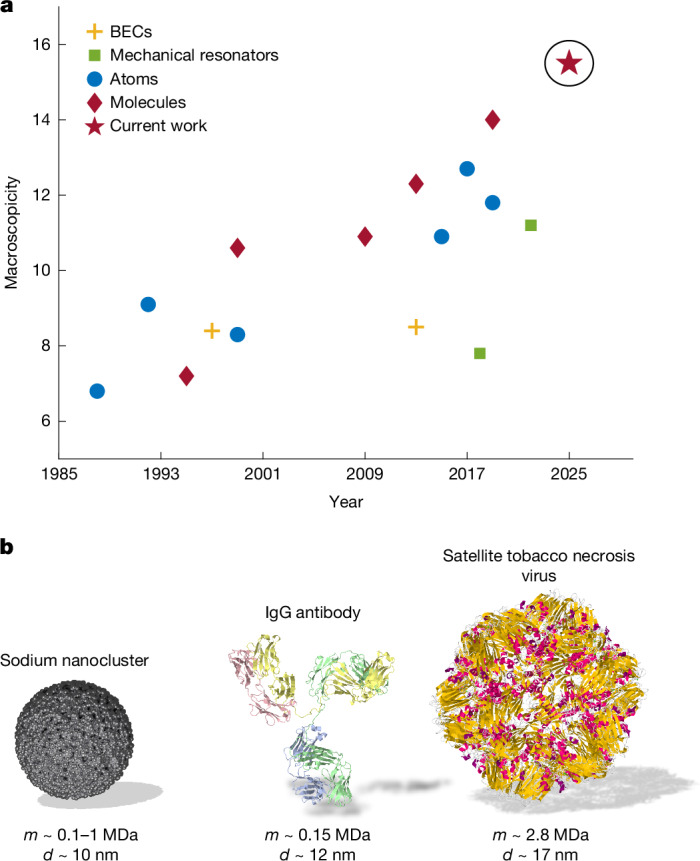
Macroscopic quantum systems and comparison. **a**, Macroscopicity values of selected quantum experiments. Blue circles represent atom interferometry; red diamonds represent molecule interferometry; orange crosses represent Bose–Einstein condensates (BECs); green squares represent mechanical resonators; and red star represents sodium nanoclusters in this study, with *μ* = 15.5. Reference data are taken from refs. ^10,11,31^ and explained in the Supplementary Information. **b**, Visualization of size and complexity. The sodium clusters studied here behave as quantum particles at about 0.2 MDa and show high contrast up to the MDa regime. The number of atoms and their mass are compatible with those of large proteins and small viruses (from protein database^53^).

Every successful demonstration of quantum interference falsifies a generic class of minimally invasive, macrorealistic modifications of quantum theory. To obtain the macroscopicity *μ*, all raw experimental data are used to narrow down the parameter space of these models by Bayesian updating, as explained in ref. ^31^. This requires a quantitative model for the outcome probabilities in the presence of macrorealistic modifications^46^. Any experimental imperfection and all decoherence processes are attributed to the macrorealistic modification and will therefore only decrease the macroscopicity (Methods). From our data, we obtain the value *μ* = 15.5, which surpasses the previous record^11^ by an order of magnitude, as shown in Fig. 4a.

The main motivation for this line of research is to explore the quantum-classical interface bottom-up, systematically, and with all parameters under control. Our interferometer is unique in that it can accept various metals and also dielectric nanoparticles with different mass densities in the same machine. An additional factor of 100 in mass and in coherence time is conceivable in a vertical interferometer^47^. This additional factor would boost the attainable macroscopicity by six orders of magnitude in a ground-based experiment, which may open new opportunities to test the weak equivalence principle with vastly different types of matter.

On the applied side, coherent self-imaging creates a cluster density pattern in free flight, which can be shifted by external forces or directed momentum kicks. Particle-like properties, such as electric or magnetic susceptibility, can then be measured on clusters while they are propagating as delocalized waves. These measurements are complementary to explorations in physical chemistry^48–50^ and promise high force resolution.

The mass of our sodium clusters (170 kDa) already surpasses that of a coconut cadang-cadang viroid (CCCVd, 81 kDa; refs. ^51,52^), or a protein such as immunoglobulin G (IgG, 150 kDa; ref. ^53^). In the next generation of experiments, it is anticipated to approach the MDa mass range of small viruses, such as the satellite tobacco necrosis virus, shown in Fig. 4b.

Although realizing quantum superpositions with these massive bio-nanomaterials still demands marked advancements in beam preparation, coherent manipulation and detection technologies, recent progress in the generation^54,55^, in tools for coherent photodepletion^56^ and in detection of beams of massive biomolecules^57^ suggests that these challenges will also be solved.

## Methods

### Quantum and classical model

The theory of Talbot–Lau interference is best formulated in phase space using the Wigner–Weyl representation of quantum mechanics^42^. This framework can account for incoherent particle sources, phase and absorption gratings, and all laser-induced photophysical effects, as well as any relevant decoherence process. It also allows for a direct comparison between the predictions of quantum and classical mechanics within the same formalism and set of assumptions.

For a cluster with mass *m* and longitudinal velocity *v*_*z*_, the probability of being detected behind the interferometer can be written as a Fourier series in the transverse position *x*_3_ of G_3_:

1 $$S({x}_{3})=\mathop{\sum }\limits_{{\ell }=-\infty }^{\infty }{S}_{{\ell }}\exp \left({\rm{i}}\frac{2{\rm{\pi }}{\ell }}{d}{x}_{3}\right).$$

In a symmetric setup with equal grating separations *L* and periods *d*, the Fourier coefficients are

2 $${S}_{{\ell }}={B}_{-{\ell }}^{(1)}(0){B}_{2{\ell }}^{(2)}\left({\ell }\frac{L}{{L}_{{\rm{T}}}}\right){B}_{{\ell }}^{(3)}(0),$$

where the Talbot–Lau coefficients ${B}_{{\ell }}^{(j)}$ of order *ℓ* for the *j*th grating still need to be determined as a function of the Talbot length *L*_T_ = *mv*_*z*_*d*^2^/*h*.

We assume that every absorbed grating photon results in the ionization of the sodium cluster. The transmission of the particle beam through a standing wave of incident laser power *P*, wavelength *λ*_L_ and Gaussian beam waist *w*_*y*_ is then characterized by the mean number of ionizing photons absorbed in each grating antinode

3 $${n}_{0}=\frac{8{\sigma }_{{\rm{ion,266}}}P{\lambda }_{{\rm{L}}}}{\sqrt{2{\rm{\pi }}}hc{w}_{y}{v}_{z}},$$

as well as by the phase shift induced by the optical dipole potential

4 $${\phi }_{0}=\sqrt{\frac{8}{{\rm{\pi }}}}\frac{{\alpha }_{266}P}{\hbar c{\varepsilon }_{0}{w}_{y}{v}_{z}}.$$

The values of the UV polarizability *α*_266_ and ionization cross-section *σ*_ion,266_ are mass-dependent and determined further below. We can then express the Talbot–Lau coefficients as^58^

5 $$\begin{array}{l}{B}_{n}(\xi )\,=\,{{\rm{e}}}^{-{n}_{0}/2}{\left(\frac{{\zeta }_{{\rm{coh}}}-{\zeta }_{{\rm{ion}}}}{{\zeta }_{{\rm{coh}}}+{\zeta }_{{\rm{ion}}}}\right)}^{n/2}\\ \,\times {J}_{n}({\rm{sgn}}({\zeta }_{{\rm{coh}}}+{\zeta }_{{\rm{ion}}})\sqrt{{\zeta }_{{\rm{coh}}}^{2}-{\zeta }_{{\rm{ion}}}^{2}}),\end{array}$$

where the coherent phase shift and the ionization depletion are described by

6 $${\zeta }_{{\rm{coh}}}(\xi )={\phi }_{0}\sin ({\rm{\pi }}\xi )$$

7 $${\zeta }_{{\rm{ion}}}(\xi )=\frac{{n}_{0}}{2}\cos ({\rm{\pi }}\xi ).$$

For short de Broglie wavelengths, as *ξ* ≡ *L*/*L*_T_ → 0, the latter turn asymptotically into the expressions

8 $${\zeta }_{{\rm{coh}}}^{{\rm{cl}}}(\xi )={\phi }_{0}{\rm{\pi }}\xi $$

9 $${\zeta }_{{\rm{ion}}}^{{\rm{cl}}}={n}_{0}/2,$$

which appear in the classical description. It yields the same expression (equations (2)–(5)) for the signal, except that equations (6) and (7) are replaced by equations (8) and (9).

In our setup, both the quantum and the classical signal are well approximated by a sinusoidal with fringe visibility *V* = 2|*S*_1_|/*S*_0_. We average the predicted signal over the measured velocity and mass distributions, accounting for the mass dependence of both the polarizability and the ionization cross-section.

### Macroscopicity assessment

To assess the macroscopicity of the demonstrated quantum superposition, it is necessary to calculate how the predicted interference signal is affected by the class of minimal macrorealist modifications (MMM) of quantum mechanics^10^. These are parameterized by the classicalization time scale *τ*_e_, and by the momentum spread *σ*_q_ and spatial spread *σ*_s_ of a phase space distribution. The greater the value of *τ*_e_, the larger the scales at which the quantum superposition principle still holds.

For our symmetric Talbot–Lau setup, the impact of an MMM is accounted for by multiplying the Fourier coefficients (equation (2)) by

10 $$\begin{array}{l}{R}_{{\ell }}\,=\,\exp \,[-2\sqrt{\frac{2}{{\rm{\pi }}}}{\left(\frac{3{\hbar }m}{{R}_{{\rm{c}}{\rm{l}}}{{\sigma }}_{{\rm{q}}}{m}_{{\rm{e}}}}\right)}^{2}\frac{L}{{v}_{z}{\tau }_{{\rm{e}}}}\\ \,\times \,{\int }_{0}^{{\rm{\infty }}}{\rm{d}}z\,{{\rm{e}}}^{-{z}^{2}/2}{j}_{1}^{2}\left(\frac{{R}_{{\rm{c}}{\rm{l}}}{{\sigma }}_{{\rm{q}}}}{{\hbar }}z\right)\,f\,\left(\frac{{\ell }d{{\sigma }}_{{\rm{q}}}L}{{\hbar }{L}_{{\rm{T}}}}z\right)]\end{array}$$

with *R*_cl_ the radius of the spherical clusters, *m*_e_ the electron mass, *j*_1_ a spherical Bessel function and *f*(*x*) = 1 − Si(*x*)/*x* involving the sine integral^10^. The dependence on *σ*_*s*_ can be neglected for this setup. The mean count rate is unaffected by MMM since *R*_0_ = 1.

The macroscopicity is obtained by using the raw experimental data ${\mathcal{C}}$ (cluster counts at given grating shift *x*_3_ and grating powers) for a Bayesian test of the hypothesis that MMM holds with a classicalization time no greater than *τ*_e_ (ref. ^31^). Bayesian updating yields the posterior probability distribution $p({\tau }_{{\rm{e}}}| {\mathcal{C}},{{\sigma }}_{{\rm{q}}})$ of the classicalization time *τ*_e_, starting from Jeffreys’ prior, by using the likelihoods obtained by incorporating equation (10) in the detection probability *S*(*x*_3_) (ref. ^46^). The lowest 5% quantile *τ*_m_(*σ*_q_) of the posterior distribution then determines the macroscopicity as $\mu =\mathop{\mathrm{max}}\limits_{{{\sigma }}_{{\rm{q}}}}({\log }_{10}({\tau }_{{\rm{m}}}({{\sigma }}_{{\rm{q}}})/1{\rm{s}}))$.

In our case, a total number of 3,895 data points yield a distribution very well approximated by a Gaussian (Kullback–Leibler divergence 1.27 × 10^−3^) whose 5% quantile *τ*_m_ = 2.84 × 10^15^ s (maximized at *ħ*/*σ*_q_ = 10  nm) remains constant to three decimal places after 3,280 data points. This indicates that sufficient data were recorded and that the distribution is independent of the prior. The resulting macroscopicity is *μ* = 15.45.

### Cluster beam

Large sodium clusters are generated in a custom-built aggregation chamber, inspired by earlier work^32,59^. The sodium is evaporated at 650–700 K into a cold mixture of argon and helium at a liquid nitrogen temperature of 77 K and pressure of less than 1 mbar. The resulting distribution covers masses beyond 1 MDa and velocities between 120 m s^−1^ and 170 m s^−1^. The clusters exit through a 5-mm aperture and pass three differential pumping stages before they reach the interferometer (Supplementary Information).

Two horizontal collimation slits *d*_H1,H2_ = 0.5 mm spaced by 1.8 m facilitate the alignment of the grating yaw angles perpendicular to the molecular beam with a precision of about 200 μrad. Two vertical collimation slits *d*_V1_ = 0.5 mm and *d*_V2_ = 1 mm, spaced by 2.2 m, confine the beam height and ensure good overlap with the standing light wave. This also reduces the influence of gravitationally induced phase averaging.

### Photophysics

The optical polarizability *α*_266_, absorption cross-section *σ*_abs,266_ and ionization potential *E*_i_ depend on the size, mass and purity of the cluster. They determine transmission, the maximal matter-wave phase shift *ϕ*_0_ and the mean number of absorbed photons *n*_0_ in the antinodes of the grating. Photophysics^60^ and thermodynamics^61^ of small sodium clusters have been extensively studied, and the preparation of particles up to 1 MDa has been demonstrated before^59^. However, the mass-selected UV polarizability has not been known. Here, we use the high-contrast fringe patterns of clusters between 0.4 MDa and 1 MDa to determine it in a mass range for which the classical and quantum models predict the same visibilities. We derive a value of *α*_266_/atom = −4π*ε*_0_ × (4.5 ± 0.5) Å^3^ (Supplementary Information), which is consistent with the experiments and the quantum model for *m* = 100–200 kDa.

The photo-ionization cross-section *σ*_ion,266_ is a product of the absorption cross-section *σ*_abs,266_ and the ionization yield. It determines the total transmission through the interferometer and influences the highest possible interference contrast. By measuring the mass-selected transmission of the interferometer for different grating powers, we determine an effective cross-section of *σ*_ion,266_ = (0.537 × *m* [kDa] − 1.5) × 10^−20^ m^2^ for our clusters.

### Mass selection and detection

After passing all gratings, the cluster beam is photo-ionized using 425 nm light and the cations are filtered by their *m*/*z* ratio using a quadrupole mass spectrometer. The mass filter includes guiding ion optics (Extrel) and 300 mm long quadrupole rods (Oxford Applied Research) with a diameter of 25.4 mm. The mass filter is operated at a resolution of Δ*m*/*m* = 0.32. Interference scans centred on mass *m*, therefore, involve clusters within a mass range of  ±Δ*m*/2, where the transmission function is close to rectangular shape and taken into account in our models. The mass filter was centred at 170 kDa. The underlying mass distribution, convoluted with the trapezoidal transmission, shifts the effective mass centre towards 172 kDa.

The selected cluster ions are counted by a channel electron multiplier with a conversion dynode at 10 kV. Electronic dark counts range from 15 to 100  counts s^−1^.

We must also account for the mixing of multiply charged ions with identical *m*/*z* ratios. Based on the measured work function of *W* = (2.4 ± 0.1) eV (Supplementary Information), neutral clusters with a diameter of *d*_Cl_ ~ 8  nm exhibit an ionization threshold of *E*_i_ = 2.53 eV, followed by *E*_i,+1_ = 2.88 eV and *E*_i,+2_ = 3.23 eV for subsequent ionization processes. The detection laser has a photon energy of *E*_ph_ = 2.92 eV and can generate doubly charged ions, whereas triply charged ions remain energetically out of reach.

We have selected doubly charged clusters in the detector and verified the correct cluster mass by analysing mass spectra at both low and high detection laser powers (Supplementary Information). In the antinodes of the gratings, the 266 nm light can also lead to multiply charged ions. However, this does not affect the interference pattern, because every ion is removed from the cluster beam by electrostatic deflection, independent of its charge state. Only clusters that remain neutral while passing through all gratings contribute to the final interference pattern.

### Velocity distribution

The cluster velocity distribution is determined from a time-of-flight measurement, in which we imprint a start time signal onto the cluster beam by UV photodepletion close to G_1_, and we measure the cluster arrival time behind the ionizing mass spectrometer. The time-of-flight data are corrected for the drift time inside the quadrupole, where it is slightly accelerated by the entrance voltage *U* to $v{\prime} =v+\sqrt{2eU/m}$. A convolution of a Gaussian drift time distribution and a rectangular chopper opening function is then fitted to the corrected unsmoothed data. The results are converted to a velocity distribution. We determine the average velocity and the width of the distribution from the standard deviation of the Gaussian fit.

Small variations of the mean velocity depend on the gas flow and the particle mass, and the 1*σ* width is Δ*v*/*v* = 5–7%. Time-of-flight and velocity spectra for *m*/*z* = 100 kTh clusters are shown in the Supplementary Information.

### Deep ultraviolet gratings

Up to 4 W of 532 nm light (Coherent Verdi V18) is converted to up to 1 W of 266 nm UV light by intracavity second harmonic generation (Sirah Wavetrain 2). The UV output is vertically expanded and split into three grating beams, using polarizing beam splitters and half-wave plates to regulate the power for each grating. Cylindrical lenses (*f* = 140 mm) focus the laser horizontally onto high-reflectivity (*R* = 99.5%) mirrors in vacuum to generate the standing light waves. We have observed power losses of up to 60% because of the degradation of optical components. The beam waists before the lenses are *W*_1_ × *H*_1_ = 1,130 × 620 μm^2^, *W*_2_ × *H*_2_ = 1,020 × 575 μm^2^ and *W*_3_ × *H*_3_ = 1,020 × 575 μm^2^, with Δ*H*_*i*_ = Δ*W*_*i*_ = ±50 μm. Here, *W*_*i*_ represents the 1/*e*^2^ waist radii along the molecular beam direction and *H*_*i*_ is the vertical waist. At the focus, the Gaussian beam waist is 20 μm. This small waist alleviates the alignment requirements with regard to the cluster beam tilt angle. The waist is still sufficiently large to ensure that the Rayleigh length, *z*_R_ = 4.7 mm, is an order of magnitude larger than the cluster beam width of 500 μm.

### Interferometer alignment

The surfaces of all three grating mirrors are aligned parallel to the particle beam axis, with the standing light wave along the mirror normal. The gratings exhibit three angular degrees of freedom: pitch, yaw and roll. The yaw angle, between the mirror surface and the particle beam, is adjusted to better than 200 μrad. The relative roll of the three mirrors, that is, their rotation around the axis parallel to the cluster beam is aligned to a difference less than 20 μrad. They are all stabilized with respect to the gravitational field of Earth to better than 50 μrad. The distances between the gratings are equal within 50 μm.

### Interference scans

We obtain the interference scans by measuring the number of transmitted clusters as a function of the transverse displacement of the third grating G_3_, which is moved in steps of Δ*x* = 15 nm. At each position, the mass-filtered ion signal is integrated for a time interval of up to four seconds. A sinusoidal fit to the data then provides the periodicity, phase and amplitude of the fringes. By design of first-order Talbot–Lau interferometry, the periodicity is equal to the grating period. Each visibility ${{\mathcal{V}}}_{i}$ results from a nonlinear least-squares sine fit to the raw counts and is accompanied by 1*σ* confidence bounds $({{\mathcal{V}}}_{i,{\rm{lb}}},{{\mathcal{V}}}_{i,{\rm{ub}}})$. We define side-specific absolute uncertainties ${\sigma }_{i,-}={{\mathcal{V}}}_{i}-{{\mathcal{V}}}_{i,{\rm{lb}}}$, ${\sigma }_{i,+}={{\mathcal{V}}}_{i,{\rm{ub}}}-{{\mathcal{V}}}_{i},$ and the effective symmetric uncertainty ${\sigma }_{i}=({\sigma }_{i,-}+{\sigma }_{i,+})/2$. Measurements are grouped by optical power into bins. For each bin ${\mathcal{B}}$, we compute the inverse-variance weighted mean $\mu ={\sum }_{i\in {\mathcal{B}}}{w}_{i}{{\mathcal{V}}}_{i}/{\sum }_{i\in {\mathcal{B}}}{w}_{i}$ with ${w}_{i}={\sigma }_{i}^{-2}$, and to display mild asymmetry, we also report ${\sigma }_{\mu ,-}^{-2}={\sum }_{i\in {\mathcal{B}}}{\sigma }_{i,-}^{-2}$ and ${\sigma }_{\mu ,+}^{-2}={\sum }_{i\in {\mathcal{B}}}{\sigma }_{i,+}^{-2}$. As a consistency check, we compute the reduced chi-square ${\chi }_{{\rm{red}}}^{2}$ using the same per-point uncertainties as the weights. For overdispersed bins (${\chi }_{{\rm{red}}}^{2} > 1.5$), we scale the upper and lower error bars of the mean by $\sqrt{{\chi }_{{\rm{red}}}^{2}}$. For visualization, plotted lower bounds are truncated at 0; all weighting and dispersion checks use the untruncated values.

## Online content

Any methods, additional references, Nature Portfolio reporting summaries, source data, extended data, supplementary information, acknowledgements, peer review information; details of author contributions and competing interests; and statements of data and code availability are available at 10.1038/s41586-025-09917-9.

## Supplementary information

Supplementary Information This file contains the following sections: (1) Experiment; (2) Phase averaging and decoherence; and (3) Quantitative analysis.

Peer Review file

## Data Availability

Data and code supporting the findings of this research are available at Zenodo (10.5281/zenodo.17502163). Additional data or materials used in the study can be provided upon request.
