# Supplementary material for: Probing quantum mechanics with nanoparticle matter-wave interferometry
Source: Nature. 2026 Jan 21;649(8098):866–70. doi: 10.1038/s41586-025-09917-9 (PMC12823444; doi:10.1038/s41586-025-09917-9)
Supplement: Supplementary file 1 — This file contains the following sections: (1) Experiment; (2) Phase averaging and decoherence; and (3) Quantitative analysis. [file 41586_2025_9917_MOESM1_ESM.pdf]

nature portfolio

https://doi.org/10.1038/s41586-025-09917-9

Supplementary information

Probing quantum mechanics with nanoparticle matter-wave interferometry

In the format provided by the authors and unedited

Supplementary Information:
Probing quantum mechanics with nanoparticle matter-wave interferometry

Sebastian Pedalino[1,2], Bruno E. Ramírez-Galindo[1,2], Richard Ferstl[1,2], Klaus Hornberger[3], Markus Arndt[1*], Stefan Gerlich[1]

[1]University of Vienna, Faculty of Physics, Boltzmanngasse 5, Vienna, 1090, Austria.
[2]University of Vienna, Vienna Doctoral School in Physics, Boltzmanngasse 5, Vienna, 1090, Austria.
[3]University of Duisburg-Essen, Faculty of Physics, Lotharstraße 1, Duisburg, 47048, Germany.

*Corresponding author(s). E-mail(s): markus.arndt@univie.ac.at;
Contributing authors: sebastian.pedalino@univie.ac.at;

Contents

1 Experiment **2**
1.1 Details of the setup . . . 2
1.2 Velocity distribution . . . 3
1.3 Photo-ionization and mass selection . . . 3
1.4 High-mass fringes and ultraviolet polarizability . . . 4

2 Phase averaging and decoherence **5**
2.1 Gravity and the rotation of the Earth . . . 5
2.2 Interferometer vibrations . . . 5
2.3 Collisional decoherence . . . 6
2.4 Thermal and optical decoherence . . . 6

3 Quantitative analysis **6**
3.1 Unscaled visibilities . . . 6
3.2 Macroscopicity . . . 6
3.3 Continuous Spontaneous Localization . . . 7
3.4 Gravitational decoherence . . . 8

1 Experiment

1.1 Details of the setup

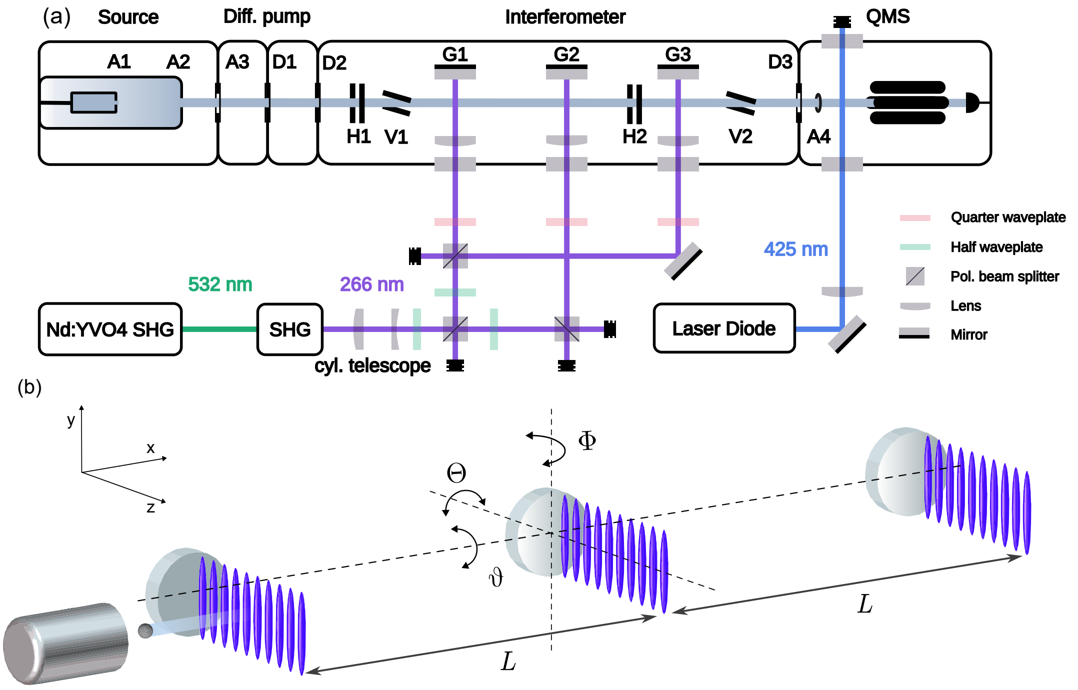

Suppl. Fig. 1: (a) Cluster beam apparatus and laser system. The D_i are apertures for differential pumping, G_i are the diffraction gratings. H and V are horizontal and vertical delimiters for alignment. The second harmonic (SHG) of the green DPSS laser light is shaped by a cylindrical telescope and focused by three cylindrical lenses to achieve a small waist ($20\,\mu$m) in the direction of the cluster beam. **(b) Schematic of the aligned degrees of freedom.** The grating angle Φ (grating yaw) is aligned with respect to the incident molecular beam, while the angle ϑ (grating roll) is aligned with respect to gravity and relative to the other gratings. The angle Θ (grating pitch) does not require critical alignment. The spacing between gratings L is set to be equal. The nanoparticle geometry in panel (b) was generated in Blender using the "FCC nanoparticle generator" asset by Ryo Mizuta Graphics.

In Suppl. Figure 1 we show details of the experimental setup, including vacuum chambers, differential pumping stages, laser system, and alignment degrees of freedom: Sodium atoms are evaporated in an effusive source with aperture (A1) and aggregate to large clusters in the cryogenic noble gas chamber. They exit through a thin aperture (A2) of 5 mm diameter into the source chamber at 4.8×10^{-3} mbar and pass another 5 mm diameter aperture (A3) into two differential pumping stages, each separated from the next by a vertical $1\times20\,\text{mm}^2$ slit, D1 and D2. These stages are pumped to 4.3×10^{-7} mbar and 5.0×10^{-8} mbar, respectively. When the cluster beam is on, the interferometer chamber reaches a pressure of 8.5×10^{-9} mbar. The detector chamber, separated by D3, is held at 8.5×10^{-8} mbar. A pair of horizontal slits, H1 and H2, collimates the cluster beam and allows us to align it to each mirror yaw angle with a precision of 200 µrad. A pair of vertical collimation slits, V1 and V2, restricts the height of the cluster beam to keep it smaller than the vertical waist of the UV gratings. This allows treating the laser beam profile as homogeneous and to minimize the sensitivity to misalignment with respect to Earth's gravity [62].

The frequency-doubled DPSS laser beam at 266 nm is divided into three parts using polarization optics. To suppress back reflections into the laser, the standing light wave is circularly polarized. The helicity of the light field does not influence the diffraction phase, as the clusters do not have any specific direction or helicity, and they come with random orientation. All gratings are mounted on motorized horizontal translation stages. G_2 and the cylindrical focusing lens, 100 mm in front of it, can additionally be vertically shifted, with nanometer resolution and over up to 1 cm in height, to compensate for the gravitational free fall of nanoparticles in different velocity classes.

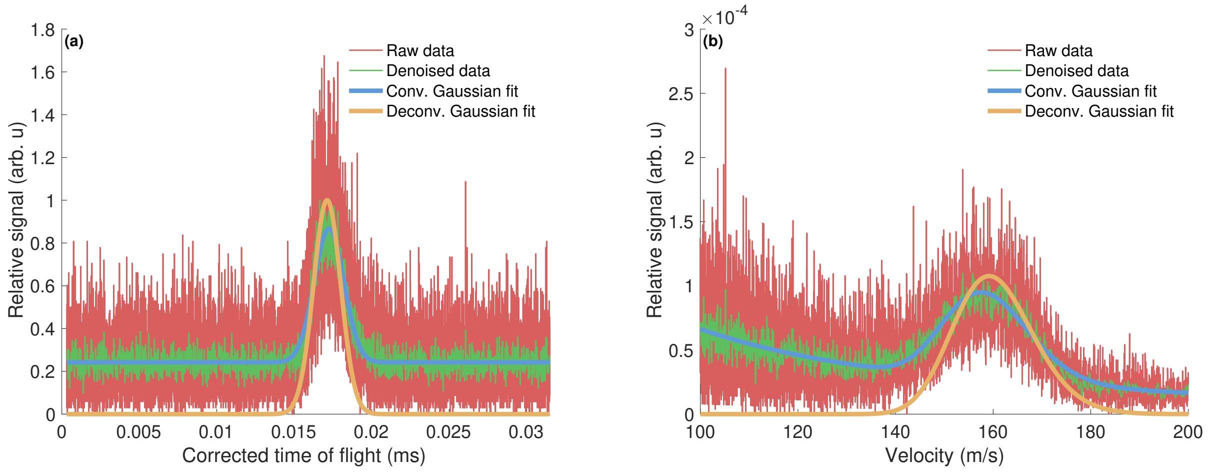

Suppl. Fig. 2: **Velocity distributions: (a)** Raw (red) and denoised (green) time-of-flight data for 100 kTh clusters. We show the Gaussian drift time (orange) and the convoluted fit to the data (blue). **(b)** Velocity distribution extracted from panel (a).

1.2 Velocity distribution

The cluster velocity is measured in a time-of-flight setting, where the start signal is given by the interruption of an ionizing UV photodepletion beam and the stop signal is the arrival time at the detector. In Suppl. Figure 2a, we show a typical time-of-flight curve, while panel (b) shows the velocity that is derived from it by transforming $f(t)\mathrm{d}t \rightarrow f(v)\mathrm{d}v$. We also account for the slight acceleration of the ions within the quadrupole mass spectrometer, which modifies the total transit time.

$$t_d = \frac{L}{v_0} + \frac{L_\mathrm{Q}}{\sqrt{v_0^2 + 2qU/m}}. \tag{1}$$

Here, $L = (2.74 \pm 0.03)\,\mathrm{m}$ is the neutral drift length in the interferometer, $L_\mathrm{Q} = (0.30 \pm 0.05)\,\mathrm{m}$ is the length of the mass filter, and $U \simeq 9\,\mathrm{V}$ the acceleration voltage in the filter. The finite opening time of the photodepletion laser is accounted for by fitting a convolution of the opening function and a Gaussian time distribution to the unsmoothed data. The maximum of the data after denoising is used as an initial guess for the fit. In several different runs, we find a mean velocity around 160 m/s, and a full width at half maximum (FWHM) of typically 10 m/s, see Suppl. Figure 2. In every run, the velocity is reconfirmed.

1.3 Photo-ionization and mass selection

Sodium has a low work function ($W = 2.75\,\mathrm{eV}$) [63], which allows for sequential photon absorption and stepwise ionization. This process continues until the ionization energy of the cluster in charge state z exceeds the photon energy $E_\gamma = h\nu$:

$$E_i[eV] = W + \frac{e(\alpha + z)}{4\pi\epsilon_0 r}. \tag{2}$$

The correction factor $\alpha \simeq 3/8$ accounts for surface effects [64].

Clusters of lower density ρ have a larger radius r and higher charge numbers can be attained. Sodium clusters with $\rho = 980\,\mathrm{kg\,m^{-3}}$ and 200 kDa can therefore be ionized manifold for $\lambda = 266\,\mathrm{nm}$. This does not affect the function of our photo-depletion gratings, as every ion is removed from the beam, independent of its charge state. However, it must be avoided in the detection process to avoid charge mixing.

Additionally, the detected signal S is influenced by the dependence of the work function on temperature T_cl and surface purity. To account for such effects, we have measured the cluster work function W under conditions identical to those prevailing in the interference experiments, using wavelengths between $430 - 504\,\mathrm{nm}$ and Fowler's law to analyze the data [65] :

$$\ln\left[\frac{S}{T_\mathrm{cl}^2}\right] = B + \ln\left[\mathrm{Li}_2\left(\frac{h\nu - W}{k_B T_\mathrm{cl}}\right)\right]. \tag{3}$$

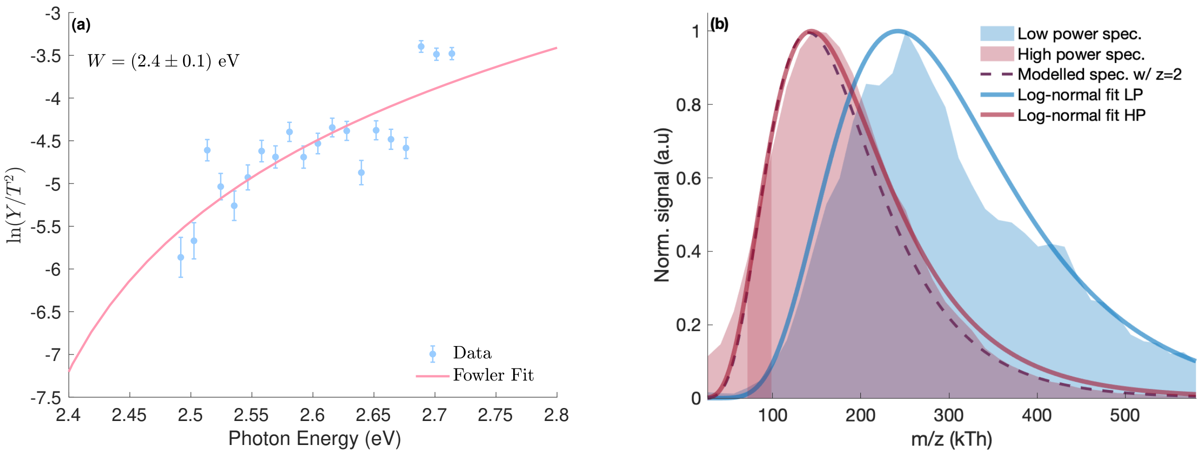

Suppl. Fig. 3: **(a) Na-Cluster ion signal as function of the photon energy.** Clusters with a diameter of $d \sim 8$ nm were ionized by the light of an optical parametric oscillator (Ekspla OPO, 100 Hz, 5 ns, $E \simeq 200-400\,\mu$J) The data (blue circles) are analyzed using a Fowler fit, shown as a solid red line. We fit $W = (2.4 \pm 0.1)$ eV. **(b) Mass spectra of sodium clusters**. The cluster beam is photo-ionized with 425 nm light at 15 mW (blue area) and 650 mW (red area). At low laser power, the clusters are only singly ionized, while they dominate in the 2+ state at high laser power. For interference experiments we use the left flank of the high-power spectrum. A model based on a log-normal size distribution (red and blue solid line) and size-dependent ionization probability reproduces the experimental data very well (dashed line). The spectrum corresponds to the interference scans in the main text, with the center mass marked as a dark vertical stripe at $m/z = 85$ kTh.

Here, B is a material-specific fit constant, and the polylogarithmic function is

$$\mathrm{Li}_2(z) = -\int_0^z \frac{\ln(1-t)}{t}\mathrm{d}t \quad \text{for } |z| \leq 1. \tag{4}$$

Suppl. Figure 3a shows the experimental data along with a Fowler fit. Assuming the cluster temperature to be in equilibrium with the collisional bath in the source, the fit yields a work function of $W = (2.4 \pm 0.1)$ eV. This low value of W is consistent with some oxygen contamination. Based on this value, we expect to find at most doubly charged clusters when using a detection laser wavelength of $\lambda = 425$ nm.

To verify this, we have first recorded a mass spectrum at low laser power $P_\mathrm{L} = 15$ mW. This avoids double ionization across the entire accessible mass range and allows us to identify the smallest and the highest masses in the spectrum, see Suppl. Figure 3b. To increase the signal, we then switch to $P_\mathrm{L} = 650$ mW, where double ionization dominates while triple ionization remains energetically inaccessible. In the low-power spectrum (blue) of panel (b), singly ionized clusters with masses below 100 kDa are essentially absent. Therefore, the left flank must be due to doubly ionized clusters. This allows us to assign $m/z \lesssim 100$ kTh in the high-power spectrum (red) to be confidently assigned to twice the measured m/z value. The dashed line in the same panel shows a modeled high-power spectrum with a maximum charge state of +2, based on the assumption that the low-power spectrum is singly charged. It aligns well with our observed high-power spectrum.

1.4 High-mass fringes and ultraviolet polarizability

The grating separation of our Talbot-Lau interferometer, $L = 0.983\,\mathrm{m} > L_T = d^2/\lambda_\mathrm{dB}$ defines the smallest usable de Broglie wavelength and thus the greatest cluster mass for the given velocity of 160 m/s. For shorter wavelengths, wave mechanics transitions into geometric optics. In this limit the interaction with grating G_2 is then fully determined by the quantities (see Methods)

$$\zeta_\mathrm{coh}(\xi) \simeq \phi_0 \pi \xi. \tag{5}$$

$$\zeta_\mathrm{ion} \simeq n_0/2 \tag{6}$$

which coincides with the classical prediction. The value of n_0 can be determined by the grating transmission, leaving only ϕ_0 as a free parameter. It depends only on the product of laser power divided by velocity, the total cluster polarizability, and $\xi \equiv L/L_T \propto 1/m$. This means that $\zeta_\mathrm{coh}(\xi)$ becomes effectively independent of mass if the total polarizability scales linearly with m and the velocity is independent of it.

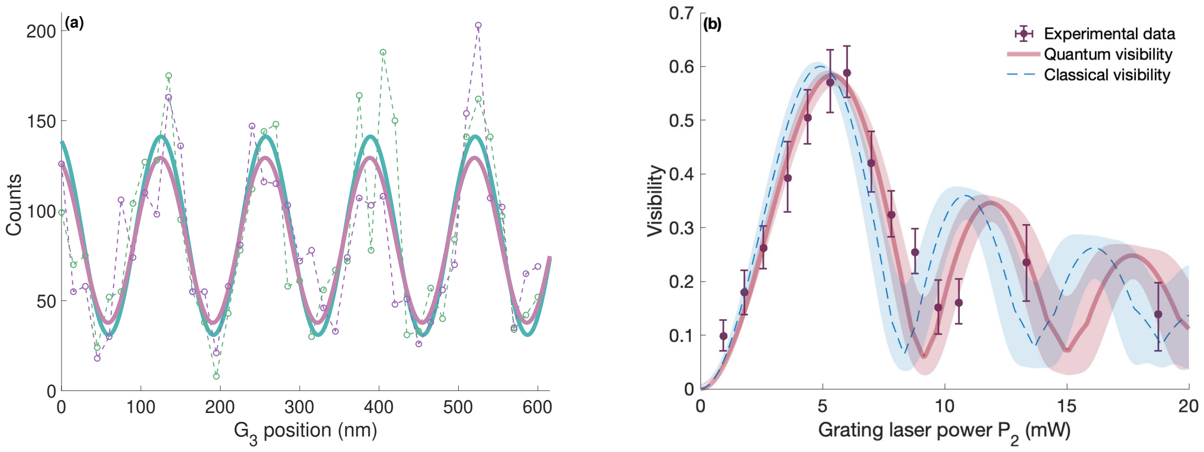

Suppl. Fig. 4: **(a) Fringe pattern of high-mass sodium clusters.** The contributing masses range between 0.4 MDa and 1.0 MDa. The grating laser powers are $P_1 = P_3 = (60\pm5)$ mW and $P_2 = (6.2\pm0.5)$ mW. Sinusoidal fits to the experimental data (circles) identify a visibility of $V = 0.66 \pm 0.09$ (green line) and $V = 0.56 \pm 0.10$ (red line). **(b) Fringe visibility as a function of the grating laser power in G_2.** $P_{1,3}$ and the masses are like in panel (a). The experimental data (purple circles) are equally well described by the quantum (red solid line) and the classical (blue dashed line) prediction. The shaded areas represent the effect of known uncertainties in the cluster velocity in the model. We extract the polarizability per atom from a fit to the model and find $\alpha_{266}/\text{atom} = -4\pi\varepsilon_0 \times (4.5 \pm 0.5)\,\text{Å}^3$.

A fit of the model to the measured fringe visibilities therefore provides a robust method for extracting the optical polarizability per atom without the need to know the precise mass distribution and without needing to distinguish between quantum and classical effects.

We find a fringe visibility of $V \simeq 0.66 \pm 0.09$ for clusters within $0.4 - 1.0$ MDa and use the G_2 power dependence $V(P_2)$ to extract the polarizability $\alpha_{266}/\text{atom} = -4\pi\varepsilon_0 \times (4.5 \pm 0.5)\,\text{Å}^3$. This value is applied to model the interference scans of all clusters, both quantum mechanically and using classical theory.

2 Phase averaging and decoherence

2.1 Gravity and the rotation of the Earth

The interference fringes are shifted in the presence of the Earth's gravity and rotation. For an appropriate choice of the grating roll angles, both accelerations cancel to first order [66]. We estimate a contrast reduction factor $R > 0.99$ from these effects for the velocity distribution in our experiment.

2.2 Interferometer vibrations

Grating vibrations can reduce the fringe visibility by

$$R_0 = |\langle \exp\left[i2\pi(\Delta x_1 - 2\Delta x_2 + \Delta x_3)/d\right]\rangle|, \tag{7}$$

where $\langle\cdot\rangle$ indicates the ensemble average over all grating shifts.

When sinusoidal vibrations across a broad frequency spectrum add randomly varying amplitudes and frequencies, one can model the displacements $\Delta x_i, (i = 1, 2, 3)$ as a stochastic variable with normal distribution. If the root mean squared amplitude Δx_{RMS} is the same in all three gratings, the contrast reduction factor is

$$R_V = \left|\exp\left(-8\pi^2 \Delta x_{\text{RMS}}^2/d^2\right)\right|. \tag{8}$$

In order to minimize vibrations, all gratings are mounted on motorized stages on a single 160 kg Invar bar that is suspended by four piano wires from an aluminum gallows supported by four springs. Additionally, Teflon balls between stacks of steel and eddy current brakes contribute to isolation and damping. The entire vacuum chamber is mounted on an optical table (resonance frequency 2 Hz) which floats on pneumatic feet [66].

We monitor the vibrations using a tri-axial accelerometer (PCB 356M98) on the Invar bar and find the vibration amplitude to lie below the accelerometer noise floor. This level of isolation is compatible with the

observed fringe visibility: An average grating vibration as small as $\Delta x_{\mathrm{RMS}} \simeq 7\,\mathrm{nm}$ could by itself explain the observed total contrast reduction factor – even in the absence of any other decoherence or phase averaging mechanism.

2.3 Collisional decoherence

The interferometer chamber is pumped by four turbo molecular pumps (Pfeiffer Hi-Pace 700), two combined ion getter and titanium sublimation pumps (Varian VacIon Plus 300 Combination) as well as two non-evaporable getter pumps (SAES Capacitorr Z1000). It is separated from the source and detection chamber by three differential pumping stages. This ensures a base pressure of 4×10^{-9} mbar and below 9×10^{-9} mbar during the operation of the cluster source.

Collisions can decohere the interference contrast if the clusters remain within the detection angle. We then expect a reduction factor $R_c = \exp(-2\Gamma_c T)$ that scales with the time in the interferometer, $2T$, and the collision rate Γ_c, which is dominated by van der Waals interactions [67, 68]:

$$\Gamma_c \approx \frac{8p_g}{k_{\mathrm{B}}T_g}\left(\frac{C_6^2 v_g^3}{\hbar^2}\right)^{1/5}. \tag{9}$$

Here p_g, T_g, and v_g are the pressure, temperature, and most probable velocity of the background gas, respectively. The coefficient C_6 can be estimated using the Slater-Kirkwood formula [68].

$$C_6 \approx \frac{3}{2}\frac{e\hbar}{\sqrt{m_e}(4\pi\varepsilon_0)^2}\frac{\bar{\alpha}_g\bar{\alpha}}{\sqrt{\bar{\alpha}_g/N_g}+\sqrt{\bar{\alpha}/N}} \tag{10}$$

The expected contrast reduction at our measured pressure $p_g = 9 \times 10^{-9}$ mbar and $T_c = 300\,\mathrm{K}$ then depends on the partial pressure in the chamber at the time of the measurement. While we did not have a calibrated rest gas analyzer inside the interferometer chamber, a model distribution composed of 4.6×10^{-9} mbar (H_2O), 1.4×10^{-9} mbar (He) and 3×10^{-9} mbar (H_2) [69] for our unbaked chamber is sufficient to explain an experimental visibility reduction factor of $R_c = 0.79$. Because of its high velocity, H_2 is the most detrimental and was estimated to have a larger contribution here to give a lower bound for R_c.

For this estimate we use the static polarizability of bulk sodium $\bar{\alpha}/N_{\mathrm{atoms}} = 4\pi\varepsilon_0 \times 9.4\,\mathrm{\AA}^3$ and one valence electron per atom $N = N_{\mathrm{atoms}}$. The rest gas polarizabilities and valence numbers are $\bar{\alpha}_g = 4\pi\varepsilon_0 \times 0.8\,\mathrm{\AA}^3$, $N_g = 2$ for H_2, $\bar{\alpha}_g = 4\pi\varepsilon_0 \times 1.5\,\mathrm{\AA}^3$, $N_g = 8$ for H_2O and $\bar{\alpha}_g = 4\pi\varepsilon_0 \times 0.2\,\mathrm{\AA}^3$, $N_g = 2$ for He [70].

2.4 Thermal and optical decoherence

In general, absorption, scattering, and emission of thermal radiation can reduce the cluster interference contrast [71]. However, for the internal temperature (ca. 80 K) of our clusters, we do not expect any observable effect related to emission [72]. Similarly, Rayleigh scattering at the optical gratings and evaporation of individual sodium atoms can be neglected [73].

3 Quantitative analysis

3.1 Unscaled visibilities

In Suppl. Figure 5, we present the unscaled visibilities as a function of the G_2 grating laser power, comparing the results to predictions from both the classical and quantum models. The visibilities were derived from sinusoidal fits of the raw interference scans, as described in the Methods.

3.2 Macroscopicity

In Suppl. Figure 6 we provide the final posterior of the classicalization time distribution (blue line), which determines the macroscopicity reached in the experiment (arrow). Jeffreys' prior (dashed line) and intermediate posteriors (black lines) are shown as well.

In Figure 4a of the main text, we compare macroscopicity values for a selection of quantum experiments. They were provided by [74–77] or estimated in the original works, as summarized in Suppl. Table 1.

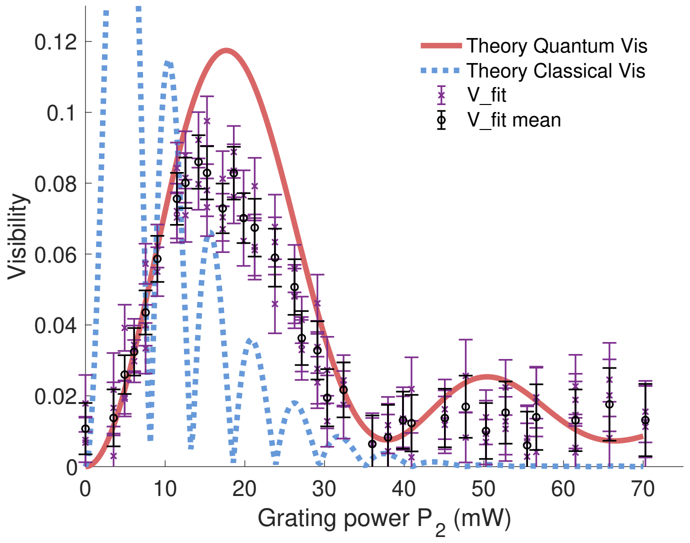

Suppl. Fig. 5: We show the measured raw visibilities for individual measurements (purple crosses) and averaged for individual laser powers (open black circles) together with the unscaled classical (blue dashed line) and quantum fringe visibility (red line) as a function of laser power in G_2 for $m_0 = 172\,\mathrm{kDa}$.

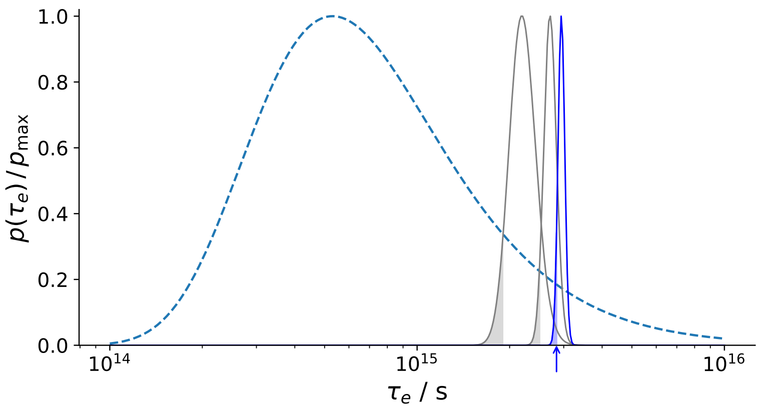

Suppl. Fig. 6: **Probability distributions $p(\tau_e)$ of the classicalization time.** The dashed line gives Jeffreys' prior, which is taken as a starting point. Bayesian updating yields posterior distributions from the recorded particle counts by means of the MMM likelihoods, calculated for the different lateral positions x_3 and grating powers P_2 with $\hbar/\sigma_q = 10\,\mathrm{nm}$. The grey lines give intermediate posterior distributions after 123 counts (left) and 410 counts (right), corresponding to 4 and 10 grating scans. The blue line gives the final posterior distribution after 95 scans (3895 data points). The shaded areas indicate the 5% quantiles, finally located at $\tau_e = 2.84 \times 10^{15}\,\mathrm{s}$ (arrow). This value remains constant to three decimals as the last 15 scans are included.

3.3 Continuous Spontaneous Localization

The theory of Continuous Spontaneous Localization (CSL) is a well-studied stochastic-collapse modification of quantum dynamics [93], which was proposed to explain why macroscopic superpositions are not observed, and to resolve the measurement problem of quantum mechanics. The theory introduces a nonlinear stochastic term in the Schrödinger equation that describes the collapse of a delocalized wave function to a small Gaussian region, whose width is given by the localization length scale r_c. The collapse rate scales quadratically with the mass m of the particle (for superpositions exceeding the particle size), and is governed by the rate parameter λ. Adler proposed that $\lambda \simeq 10^{8\pm1}\,\mathrm{Hz}$ and $r_c \simeq 100\,\mathrm{nm}$ for CSL to be a viable solution to the measurement problem [94].

The values proposed by Adler have been largely ruled out by non-interferometric measurements [95]. In particular, the search for spontaneous photon emission induced by collapse models at the Gran Sasso underground laboratory yielded the strong bound $\lambda < 5 \times 10^{-13}\,\mathrm{Hz}$ at $r_c = 100\,\mathrm{nm}$ [96]. Still, it is useful to see to what extent matter-wave interference experiments constrain the CSL parameters, because only those experiments probe the quantum state of delocalized particles.

Experiment type	Year	Macroscopicity	Ref.
Atoms	1988	6.8	[78]
	1992	9.1	[79]
	1999	8.3	[80]
	2015	10.9	[81]
	2017	12.4	[82]
	2019	11.8	[83]
BECs	1997	8.4	[84]
	2013	8.5	[85]
Mech. Resonators	2018	7.8	[86]
	2022	11.2	[87]
Molecules	1995	7.2	[88]
	1999	10.6	[89]
	2009	10.9	[90]
	2013	12.3	[91]
	2019	14.0	[92]
Metal Clusters	2025	15.5	this work

Suppl. Table 1: References for the macroscopicity values of selected quantum experiments values shown in Figure 4a of the main text.

Our experiment sets new limits on genuine quantum tests of the CSL model, as shown in Figure 7. The pink area represents the limits of our present work, while the blue area corresponds to KDTLI [97] and LUMI [92] interferometry using organic molecules. The analysis is based on the the theory in [98] describing the impact of CSL on Talbot-Lau interferece. Adler's values [94] of the collapse rate λ at localization lengths $r_c = 100\,\mathrm{nm}$ and $r_c = 1\,\mu\mathrm{m}$ are now largely excluded also in a interferometric test, but still compatible with the error bar.

3.4 Gravitational decoherence

A number of modifications of the Schrödinger equation have been proposed that lead to spatial decoherence of a delocalized massive particle due to the impact of gravity. Most notably, the Diósi model (or Diósi–Penrose model), which is based on an assumed gravitational self-interaction of the superposition state, adds the incoherent term [99]

$$\mathcal{L}\rho = -\frac{G}{2\hbar}\int \frac{d^3r d^3r'}{|\mathbf{r}-\mathbf{r}'|}[f(\mathbf{r}-\hat{x}),[f(\mathbf{r}'-\hat{x}),\rho]] \tag{11}$$

to the von Neumann equation of the center-of-mass dynamics. Here G is the gravitational constant and $f(\mathbf{r}) = 3m\Theta(r-R_0)/(4\pi R_0^3)$ is the mass density of the particle, smeared out over the length scale R_0 to avoid unphysical heating rates.

Other models lead essentially to the same form of gravitational decoherence and can thus be seen as in independent motivations: (i) The theory of Kafri Taylor, and Milburn model treats gravity as a classical measurement-and-feedback channel [100] yielding, in a linearized approximation, position decoherence that is "consistent with Diósi's model". (ii) The theory by Oppenheim [101] develops a general framework for hybrid classical-quantum dynamics, with the aim of keeping the gravitational space-time dynamics classical when coupling it consistently to quantum particles acting as mass sources. In the nonrelativistic limit, and assuming a Netwonian scaling $\propto 1/|x-y|$ of the decoherence kernel one ends up with the form of the Diosi master equation. (iii) The entropic model of gravity by Carney et al. [102] models gravity as an entropic force produced by mediator qubits, located at a lattice with constant a, whose energies are determined by the particle positions. The characteristic decoherence rate (66) in [102] has again the same form (up to a factor of order unity) as the Diósi–Penrose rate, with the smearing scale R_0 replaced by the lattice constant a. (iv) The Schrödinger-Newton equation [103], where the wave function contributes energy through a gravitational interaction with its "mass density" $m|\psi|^2$, can be made non-signaling by adding stochastic terms, which again yields a master equation of the form (11) [104].

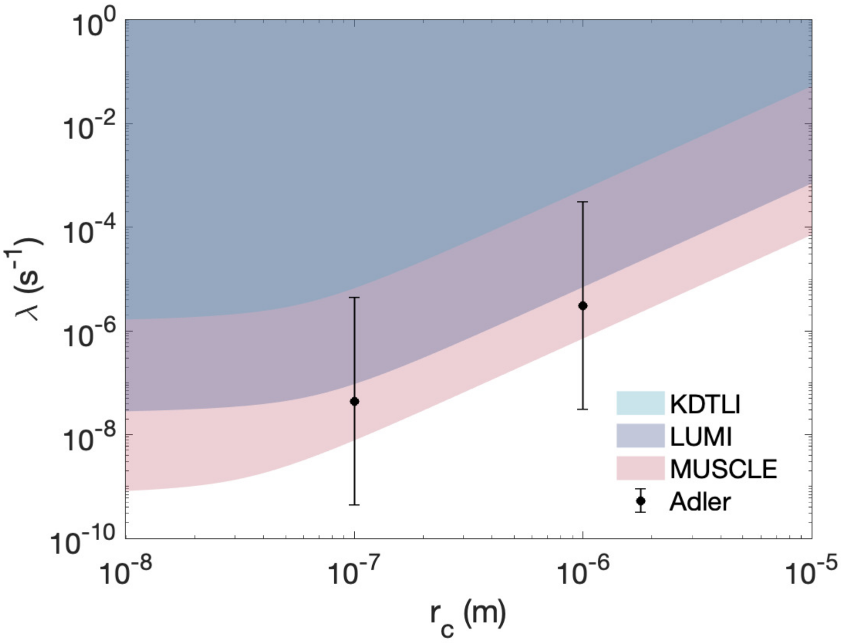

Suppl. Fig. 7: Experimental constraints on the localization rate λ and the localization length r_c of the Continuous Spontaneous Localization model. The pink area represents the parameter set excluded by our present interferometric work, based on a demonstrated visibility of no less that 78% of the ideal quantum prediction for sodium clusters of mass $m = 172$ kDa and velocity of $v = 160$ m/s. The blue area corresponds to previous constraints from KDTLI [97] and LUMI [92] interferometry with organic molecules. Adler's proposed values [94] are ruled out to a large extent, but remain marginally compatible within their bounds. Stricter constraints from non-interferometric tests [96] are not shown.

The present experiment does not test the Diósi–Penrose model because the predicted decoherence is too small. The gravitational decoherence rate predicted by (11) is

$$\Gamma_{\max} \simeq \frac{6Gm^2}{\hbar R_0}. \tag{12}$$

Given the interference time of about 12 ms, one would be sensitive to this gravitational decoherence only for $R_0 < 10^{-21}$ m, much smaller than the natural scale, as given by the size of an atomic nucleus. At the same time, non-interferometric measurements show that the model is empirically consistent only with R_0 being more than ten orders of magnitude greater [96]. To become sensitive to this scale, future experiments will have to demonstrate the spatial delocalization of a 10^8 Da particle over ten seconds, or 10^9 Da over 100 ms.

References

[62] Pedalino, S., Ramirez Galindo, B., Sousa, T., Fein, Y.F., Geyer, P., Gerlich, S., Arndt, M.: Experimental challenges for high-mass matter-wave interference with nanoparticles. SPIE, Quant. Sens., Imag. and Prec. Metrol. **12447**, 1–10 (2023) https://doi.org/10.1117/12.2657260

[63] Wong, K., Tikhonov, G., Kresin, V.V.: Temperature-dependent work functions of free alkali-metal nanoparticles. Phys. Rev. B **66**, 125401 (2002) https://doi.org/10.1103/PhysRevB.66.125401

[64] De Heer, W.A.: The physics of simple metal clusters: Experimental aspects and simple models. Rev. Mod. Phys. **65**, 611–676 (1993) https://doi.org/10.1103/RevModPhys.65.611

[65] Fowler, R.H.: The analysis of photoelectric sensitivity curves for clean metals at various temperatures. Phys. Rev. **38**, 45–56 (1931) https://doi.org/10.1103/PhysRev.38.45

[66] Fein, Y.Y., Kiałka, F., Geyer, P., Gerlich, S., Arndt, M.: Coriolis compensation via gravity in a matter-wave interferometer. New J. Phys. **22**, 033013 (2020) https://doi.org/10.1088/1367-2630/ab73c5

[67] Hornberger, K., Uttenthaler, S., Brezger, B., Hackermüller, L., Arndt, M., Zeilinger, A.: Collisional decoherence observed in matter wave interferometry. Phys. Rev. Lett. **90**, 160401 (2003) https://doi.org/10.1103/PhysRevLett.90.160401

[68] Kiałka, F., Fein, Y.Y., Pedalino, S., Gerlich, S., Arndt, M.: A roadmap for universal high-mass matter-wave interferometry. AVS Quant. Sci. **4**, 020502 (2022) https://doi.org/10.1116/5.0080940

[69] Dobrozemsky, R.: Experience with a Computer Program for Residual Gas Analyzers. Journal of Vacuum Science and Technology **9**(1), 220–223 (1972) https://doi.org/10.1116/1.1316559

[70] Olney, T.N., Cann, N.M., Cooper, G., Brion, C.E.: Absolute scale determination for photoabsorption spectra and the calculation of molecular properties using dipole sum-rules. Chemical Physics **223**(1), 59–98 (1997) https://doi.org/10.1016/S0301-0104(97)00145-6

[71] Hackermüller, L., Hornberger, K., Brezger, B., Zeilinger, A., Arndt, M.: Decoherence of matter waves by thermal emission of radiation. Nature **427**, 711–714 (2004) https://doi.org/10.1038/Nature02276

[72] Schäfer, J., Stickler, B.A., Hornberger, K.: Decoherence of dielectric particles by thermal emission. Physical Review Research **6**(4), 043307 (2024) https://doi.org/10.1103/PhysRevResearch.6.043307

[73] Schäfer, J., Stickler, B.A., Hornberger, K.: Desorption-Induced Decoherence of Nanoparticle Motion. arXiv (2025). https://doi.org/10.48550/arXiv.2503.05475

[74] Nimmrichter, S., Hornberger, K.: Macroscopicity of Mechanical Quantum Superposition States. Phys. Rev. Lett. **110**(16), 160403 (2013) https://doi.org/10.1103/PhysRevLett.110.160403

[75] Schrinski, B., Yang, Y., Von Lüpke, U., Bild, M., Chu, Y., Hornberger, K., Nimmrichter, S., Fadel, M.: Macroscopic Quantum Test with Bulk Acoustic Wave Resonators. Phys. Rev. Lett. **130**(13), 133604 (2023) https://doi.org/10.1103/PhysRevLett.130.133604

[76] Schrinski, B., Nimmrichter, S., Stickler, B.A., Hornberger, K.: Macroscopicity of quantum mechanical superposition tests via hypothesis falsification. Phys. Rev. A **100**(3), 032111 (2019) https://doi.org/10.1103/PhysRevA.100.032111

[77] Schrinski, B., Nimmrichter, S., Hornberger, K.: Quantum-classical hypothesis tests in macroscopic matter-wave interferometry. Phys. Rev. Res. **2**, 033034 (2020) https://doi.org/10.1103/PhysRevResearch.2.033034

[78] Keith, D.W., Schattenburg, M.L., Smith, H.I., Pritchard, D.E.: Diffraction of Atoms by a Transmission Grating. Phys. Rev. Lett. **61**, 1580–1583 (1988) https://doi.org/10.1103/PhysRevLett.61.1580

[79] Shimizu, F., Shimizu, K., Takuma, H.: Double-slit interference with ultracold metastable neon atoms. Phys. Rev. A **46**, 17–20 (1992) https://doi.org/10.1103/PhysRevA.46.R17

[80] Grisenti, R.E., Schöllkopf, W., Toennies, J.P., Hegerfeldt, G.C., Köhler, T.: Determination of Atom-Surface

van der Waals Potentials from Transmission-Grating Diffraction Intensities. Phys. Rev. Lett. **83**, 1755–1758 (1999) https://doi.org/10.1103/PhysRevLett.83.1755

[81] Kovachy, T., Asenbaum, P., Overstreet, C., Donnelly, C.A., Dickerson, S.M., Sugarbaker, A., Hogan, J.M., Kasevich, M.A.: Quantum superposition at the half-metre scale. Nature **528**, 530–3 (2015) https://doi.org/10.1038/nature16155

[82] Asenbaum, P., Overstreet, C., Kovachy, T., Brown, D.D., Hogan, J.M., Kasevich, M.A.: Phase Shift in an Atom Interferometer due to Spacetime Curvature across its Wave Function. Phys. Rev. Lett. **118**, 183602 (2017) https://doi.org/10.1103/PhysRevLett.118.183602

[83] Xu, V., Jaffe, M., Panda, C.D., Kristensen, S.L., Clark, L.W., Müller, H.: Probing gravity by holding atoms for 20 seconds. Science **366**, 745–749 (2019) https://doi.org/10.1126/science.aay6428

[84] Andrews, M.R., Townsend, C.G., Miesner, H.-J., Durfee, D.S., Kurn, D.M., Ketterle, W.: Observation of Interference Between Two Bose Condensates. Science **275**, 637–641 (1997) https://doi.org/10.1126/science.275.5300.637

[85] Berrada, T., Van Frank, S., Bücker, R., Schumm, T., Schaff, J.-F., Schmiedmayer, J.: Integrated Mach–Zehnder interferometer for Bose–Einstein condensates. Nat. Communs. **4**, 2077 (2013) https://doi.org/10.1038/ncomms3077

[86] Riedinger, R., Wallucks, A., Marinković, I., Löschnauer, C., Aspelmeyer, M., Hong, S., Gröblacher, S.: Remote quantum entanglement between two micromechanical oscillators. Nature **556**(7702), 473–477 (2018) https://doi.org/10.1038/s41586-018-0036-z

[87] Von Lüpke, U., Yang, Y., Bild, M., Michaud, L., Fadel, M., Chu, Y.: Parity measurement in the strong dispersive regime of circuit quantum acoustodynamics. Nat. Phys. **18**, 794–799 (2022) https://doi.org/10.1038/s41567-022-01591-2

[88] Chapman, M.S., Ekstrom, C.R., Hammond, T.D., Rubenstein, R.A., Schmiedmayer, J., Wehinger, S., Pritchard, D.E.: Optics and Interferometry with Na 2 Molecules. Phys. Rev. Lett. **74**, 4783–4786 (1995) https://doi.org/10.1103/PhysRevLett.74.4783

[89] Arndt, M., Nairz, O., Voss-Andreae, J., Keller, C., Zouw, G., Zeilinger, A.: Wave-particle duality of C_{60} molecules. Nature **401**, 680–682 (1999) https://doi.org/10.1038/44348

[90] Hornberger, K., Gerlich, S., Ulbricht, H., Hackermüller, L., Nimmrichter, S., V Goldt, I., Boltalina, O., Arndt, M.: Theory and experimental verification of Kapitza–Dirac–Talbot–Lau interferometry. New J. Phys. **11**, 043032 (2009) https://doi.org/10.1088/1367-2630/11/4/043032

[91] Eibenberger, S., Gerlich, S., Arndt, M., Mayor, M., Tüxen, J.: Matter–wave interference of particles selected from a molecular library with masses exceeding 10 000 amu. Phys. Chem. Chem. Phys. **15**, 14696 (2013) https://doi.org/10.1039/c3cp51500a

[92] Fein, Y.Y., Geyer, P., Zwick, P., Kiałka, F., Pedalino, S., Mayor, M., Gerlich, S., Arndt, M.: Quantum superposition of molecules beyond 25 kda. Nat. Phys. **15**, 1242–1245 (2019) https://doi.org/10.1038/s41567-019-0663-9

[93] Bassi, A., Ghirardi, G.: Dynamical reduction models. Phys. Rep. **379**(5), 257–426 (2003) https://doi.org/10.1016/S0370-1573(03)00103-0

[94] Adler, S.L.: Lower and upper bounds on CSL parameters from latent image formation and IGM heating. J. Phys. A: Math. Theor. **40**(12), 2935 (2007) https://doi.org/10.1088/1751-8113/40/12/S03

[95] Vinante, A., Carlesso, M., Bassi, A., Chiasera, A., Varas, S., Falferi, P., Margesin, B., Mezzena, R., Ulbricht, H.: Narrowing the Parameter Space of Collapse Models with Ultracold Layered Force Sensors. Phys. Rev. Lett. **125**(10), 100404 (2020) https://doi.org/10.1103/PhysRevLett.125.100404

[96] Donadi, S., Piscicchia, K., Curceanu, C., Diósi, L., Laubenstein, M., Bassi, A.: Underground test of gravity-related wave function collapse. Nat. Phys. **17**, 74–78 (2021) https://doi.org/10.1038/s41567-020-1008-4

[97] Gerlich, S., Eibenberger, S., Tomandl, M., Nimmrichter, S., Hornberger, K., Fagan, P., Tüxen, J., Mayor, M., Arndt, M.: Quantum interference of large organic molecules. Nat. Commun. **2**, 263 (2011)

[98] Nimmrichter, S., Hornberger, K., Haslinger, P., Arndt, M.: Testing spontaneous localization theories with matter-wave interferometry. Phys. Rev. A **83**(4), 043621 (2011) https://doi.org/10.1103/PhysRevA.83.043621

[99] Diósi, L.: A universal master equation for the gravitational violation of quantum mechanics. Phys. Lett. A **120**(8), 377–381 (1987) https://doi.org/10.1016/0375-9601(87)90681-5

[100] Kafri, D., Taylor, J.M., Milburn, G.J.: A classical channel model for gravitational decoherence. New J. Phys. **16**(6), 065020 (2014) https://doi.org/10.1088/1367-2630/16/6/065020

[101] Oppenheim, J., Sparaciari, C., Soda, B., Weller-Davies, Z.: Gravitationally induced decoherence vs spacetime diffusion: testing the quantum nature of gravity. Nat. Commun. **14**, 7910 (2023) https://doi.org/10.1038/s41467-023-43348-2

[102] Carney, D., Karydas, M., Scharnhorst, T., Singh, R., Taylor, J.M.: On the Quantum Mechanics of Entropic Forces. Phys. Rev. X **15**(3), 031038 (2025) https://doi.org/10.1103/y7sy-3by1

[103] Ruffini, R., Bonazzola, S.: Systems of Self-Gravitating Particles in General Relativity and the Concept of an Equation of State. Phys. Rev. **187**(5), 1767–1783 (1969) https://doi.org/10.1103/PhysRev.187.1767

[104] Nimmrichter, S., Hornberger, K.: Stochastic extensions of the regularized Schrödinger-Newton equation. Phys. Rev. D **91**(2), 024016 (2015) https://doi.org/10.1103/PhysRevD.91.024016
