# Supplementary material for: Probing quantum mechanics with nanoparticle matter-wave interferometry
Source: Nature. 2026 Jan 21;649(8098):866–70. doi: 10.1038/s41586-025-09917-9 (PMC12823444; doi:10.1038/s41586-025-09917-9)
Supplement: Supplementary file 2 — Peer Review file [file 41586_2025_9917_MOESM2_ESM.pdf]

nature portfolio

Peer Review File

Probing quantum mechanics with nanoparticle matter-wave interferometry

Corresponding Author: Professor Markus Arndt

Any redactions in this file are there to maintain patient confidentiality, the confidentiality of unpublished data, or to remove third-party material.

This file contains all reviewer reports in order by version, followed by all author rebuttals in order by version.

Version 0:

Reviewer comments:

Referee #1

(Remarks to the Author)
The paper by Pedalino et al reports on a fascinating new experimental test of the validity of the quantum superposition principle at large masses and long times. The authors use matter wave interferometry of a large sodium clusters to demonstrate high visibility interference fringes in a new size/time/mass regime of massive particles.
They have built a standing light wave Talbot-Lau interferometer of ultra-high stability and sent cold (!) nanoparticles through that interferometer, observing interference in the quantum regime of the interferometer (in a Talbot-Lau setup, there is also a regime where fringes are produced by classical means of a Moiré-type pattern). The experiment is technically highly challenging and the paper and the suppl material once more demonstrate the internationally unsurpassed level of technical expertise by the Arndt group. The technical part of the paper is very well written and the supplementary material gives an excellent documentation of all details which came to mind of the present referee. They nicely discuss known other mechanisms of loss of interference contrast, like decoherence by light scattering or black body radiation, and collisions with residual gas atoms, and give a good account of the stabilization of the interferometer, the detection scheme, and the cluster source and characterization. References are excellent and well choosen. Previous work is fully acknowledged.

While the technical part of the paper is flawless and exhausting, and the data and their analysis, including error analysis, are of very high quality, the general claim of the paper for a non-specialized audience should be strengthened and explained better and less technical and jargon-loaded. The general claim roughly formulated is: - we have tested the heart of quantum mechanics, the superposition principle, in a new regime and can exclude alternative theories a factor of 10 more sensitively than previous experiments - this is easy to grasp. But what does that actually mean? What are the alternatives which is ruled out, and how does such an alternative model predict a degrading of interference visibility? I feel that an article in Nature should meet that challenge to make it understandable to at least a physics-educated reader what is really achieved. The current paper fails at this point. The paper tries to solve this by referencing, but this approach is ineffective. The literature e.g. [30] or the review [15] to which the reader is directed, is very technical and does not help a reader who is not already a specialist in the field.
I suggest that the authors address this issue by being courageous: put one of the theories which is falsified by the new experiment in words (not equations), even if it comes at the price of being not 100% accurate for a specialist. Do something which one does normally in talks (at least one of the authors is really good at this) but which one mostly does not do in papers: give handwaving explanations even if they are only a part of the truth. Tell a story like „Theory A predicts that at a mass of XYZ gravitation on earth over a time of TTT would do ABC to the wavefunction …" OR „Theory B predicts that a timescale which is longer than XYZ, the wavefunction gradually collapses by itself without the need for any interaction by forces and fields – the extended particle shrinks to a point in position space and XYZ in momentum space. If it is in a superposition of being in a left or right slit in a double slit for longer than TTT it would do XYZ …" (please do not take these examples by word, I know that this is wrong. It is meant as an illustration of what the referee means by a handwaving, non-specialist, non-jargon, explanation).
Once such an example is formulated, one can explain what this particular theory would predict for the currently studied interference and which is the most critical experimental parameter (mass, size, time), and how the experiment falsifies that theory and why all other experiments cannot falsify that (possibly bizarre) theory.
After such an example is formulated, of course, one has to get serious and explain and introduce the macroscopicity as a

concept which makes this falsification of theories more general.

In conclusion I strongly support publication of the present work in Nature after (didactical) changes. The topic is fascinating for a general audience, the experimental work is cutting etch and the claim is very well justified by the data – it should just be explained more didactically to a non-expert audience.

Referee #2

(Remarks to the Author)
Review of manuscript 2025-06-15934

I enjoyed reading this manuscript very much. The authors present an updated forefront study of matter-wave experiments and a careful analysis thereof extending their own research program to larger and heavier particles. Specifically, they clearly demonstrate that 172 kDa gold cluster exhibit significant quantum superposition in their experiment. This significantly extends the size/mass of the objects for which matter-wave interference could be observed 7-fold. It also increases the important "macroscopicity" across different experimental approaches from approx. 14 to approx. 16.

These results should be seen in light of a long history of successful evolutions of this experiment, from C60 clusters [Nature 401, 680 (1999)] to, e.g., small macromolecules with 25 kDa [Nat. Phys. 15, 1242 (2019)]. The current progress is i) to create highly collimated beams of large metal clusters, based on the work by Haberland and others (appropriately cited in the manuscript), ii) to implement a 266 nm Talbot-Lau interferomenter, building upon own developments, e.g., Phys. Chem. Chem. Phys., 2024, 27617-27623, and iii) to implement a commercial mass-selective detection scheme.

The experimental and computational implementations, data, and analysis look valid and appropriate, statistics seem correct and – including all analysis – are well described. The abstract is good. The discussion should point out what the central "punchline" finding of this work actually is that warrants publication in a very-high-impact journal. Own previous work is not very well summarized nor cited, corresponding updates to the introduction and experimental details would be useful.

Overall, to my mind, this manuscript can almost be published as is, i.e., with updates as stated above.

However, to me this is, albeit surely an heroic effort, a somewhat "incremental" improvement over previous work by the same authors. Unless I am overseeing a truly significant qualitative improvement over previous work (see above), I suggest to publish this work in a more specialized journal.

Referee #3

(Remarks to the Author)
An important question at the forefront of modern science is if, in principle, arbitrarily large objects can exist in quantum superpositions, or if instead there is a fundamental mechanism that prevents this for sufficiently large objects. Advances in matter wave interferometry have brought this question from the realm of thought experiments into something that can be probed in the laboratory. The manuscript by Pedalino et al. presents a remarkable advance in this area, using a Talbot-Lau interferometer to demonstrate matter wave interference with nanoparticles of mass in excess of 170,000 dalton. This improves by approximately an order of magnitude over the previous record of 25,000 dalton from the same group (reference [5] in the manuscript). Not only is this result an essential landmark in its own right, but it also is a key step toward future experiments involving superpositions of massive biological objects such as antibodies or viruses.

The manuscript is well-written and clearly describes the experimental methods and analysis procedures. Figure 2 is particularly important, as it presents data together with theoretical modelling convincingly showing that results cannot be explained classically. This work is sure to be of very broad interest, and I believe that it would make an outstanding contribution to Nature, provided that the detailed comments below are addressed.

Detailed comments:

-I feel that it is important for the authors to broaden their discussion of the constraints their results place on proposed modifications to quantum mechanics, and how these constraints compare to those from other experiments. The comments below elaborate on this point.

-In the current manuscript, the discussion of such constraints focuses on the macroscopicity value. While macroscopicity is indeed a valuable and convenient metric for comparing experiments and is widely used in the literature, this same convenience also limits the information that it conveys. Many proposed modifications to quantum mechanics, such as the widely studied continuous spontaneous localization (CSL) model, are characterized by a two dimensional parameter space. For example, CSL is characterized by a rate and a correlation length, which are independent parameters. In the context of CSL, two experiments could have the same macroscopicity values but probe different parts of the two dimensional parameter space. This is because the two experiments could probe different values of the correlation length. Therefore, while the authors should certainly keep their discussion of the macroscopicity value, I believe that they should also include an exclusion plot for the CSL parameter space that compares their results to other experiments. I note that reference [5] included such a plot in Figure 3b.

-The authors state in the abstract that their record macrosopicity value provides “the most stringent exclusion limit for generic macrorealistic modifications of the Schrodinger equation to date.” For the reasons stated above, I believe that this statement would require further qualification. While I do not know whether this is the case without seeing the CSL exclusion plot requested above, it is possible that another experiment might have lower macroscopicity but actually rule out a larger area of CSL parameter space. The quoted statement in the abstract would then have the potential to cause confusion for readers, since one could then reasonably interpret the other experiment as having more stringent exclusion limits. I would suggest that the authors check this or clarify the statement in the abstract.

-In addition to decohering quantum superpositions, many proposed modifications to quantum mechanics (e.g., CSL) also would lead to spontaneous heating. Experiments that bound this spontaneous heating therefore also constrain the CSL parameter space. For completeness, I believe that the authors should compare the constraints from their work to constraints from experiments that bound spontaneous heating. A relevant reference is Vinante et al., Phys. Rev. Lett. 125, 100404 (2020). I should emphasize that these spontaneous heating constraints should not be seen as a replacement for experiments, like those in the present manuscript, that directly verify matter wave interference. But they provide an important, complementary way to probe modifications to quantum mechanics and should therefore be discussed.

-Along with CSL, it would be very valuable if the authors could comment on the constraints put by their experiment on other specific models. Relevant examples include the Kafri-Taylor-Milburn Model [Kafri, Taylor, Milburn, New. J. Phys. 16, 065020 (2014)], Oppenheim’s Postquantum Theory of Classical Gravity [Oppenheim, Phys. Rev. X 13, 041040 (2023)], and the entropic model of gravity in Carney et al., Phys. Rev. X 15, 031038 (2025).

-Is the present experiment able to test the Diosi-Penrose Model, or are there prospects for doing this with future upgrades?

-The introduction might benefit from a brief explanation of the motivation for collapse models from the point of view of the measurement problem. Reference [15] has a particularly good discussion of this point that could be cited here.

-In Figure 2(b), both the classical and quantum theory curves are scaled by the same global factor of 0.78. The authors mention that the purpose of this factor is to account for various effects that reduce contrast. However, at the moment, it is not clear to me why the same factor should be applied to the classical and quantum curves. The authors mention effects that seem to be related to interference among the list of causes of contrast reduction, for example, gravitational and rotational phase averaging and thermal or collisional decoherence. I would tend to think that such factors might have a different effect on the contrast for the classical vs. quantum case. If I am mistaken, can the authors please add a brief explanation as to why they should be the same? Otherwise, the authors should revise the factor applied to the classical case, or increase the size of the error bars on the classical case if they are not sure what the factor should be.

-In Supplement Figure 5, the description of the curves in the caption does not seem to match the legend on the plot.

-The authors use an adaptive Gaussian low-pass filter to denoise the time-of-flight data. Could the authors say more about how the “adaptive” part of the filtering algorithm works? Have the authors verified that their end results do not significantly depend on small changes in the parameters of the filter?

Referee #4

(Remarks to the Author)
This work merits publication in Nature following minor revision to address the issues outlined below. The work presented is of extremely high quality and the experiment truly groundbreaking.

The discussion of previous work is a little sparse and may not give sufficient context for this new result. The Vienna group have demonstrated a steady march of remarkable progress achieving, by my estimates, one order of magnitude increase in mass per decade. That this work is the culmination of sustained effort is not made clear. The most notable absence is the seminal 1999 paper Arndt et al. "C60 wave-particle duality" (doi:10.1038/44348). At the time of the C60 publication many believed it was simply not possible that such a complicated molecule, with many internal degrees of freedom, could exhibit matter wave interference of the centre of mass mode; the same concerns abound now about non-identical nanoparticles, and these are summarily refuted by this experiment. Making more clear the timeline of this work will also make their claims about what they may achieve in the future (line 202) more believable.

A more complete timeline might include:
- 1994, 39 Da: Clauser et al. - K atoms [CITED]
- 1995, 46 Da: Chapman et al. - Na2 molecules (doi:10.1103/PhysRevLett.74.4783)
- 1999, 720 Da: Arndt et al. - C60 wave-particle duality (doi:10.1038/44348)
- 2002, 840 Da: Brezger et al. - C70 (doi:10.1103/PhysRevLett.88.100404)
- 2007, ~1kDa: Gerlich et al. - Kapitza-Dirac-Talbot-Lau for polarizable molecules [CITED]
- 2019, 25kDa: Fein et al. - Organic molecules beyond 25 kDa [CITED]
- 2025, 170kDa, : Current work - Extension to metal nanoparticles

Additionally, the manuscript appears to under-cite the development of their experimental platform. The experiment appears to use the OTIMA interferometer, yet the manuscript omits key references describing its design and capabilities, including

Haslinger et al. (2013) "A universal matter-wave interferometer with optical ionization gratings in the time domain" (doi:10.1038/nphys2542) and (less crucially) Dörre et al. (2014) "Photofragmentation beam splitters for matter-wave interferometry" (doi:10.1103/PhysRevLett.113.233001). While the current application to metal nanoparticles is novel, readers would benefit from understanding how the experimental techniques evolved and were validated in previous studies using the same apparatus.

On a more technical note, the authors claim a visibility and provide an uncertainty on this estimate. However, the confidence in this this visibility estimate depends critically on the individual uncertainties in counts i.e. the y-axis in Fig2a. Scatter in these data points is clearly evident, but is not discussed, save for a brief mention of dark counts on line 442. Without an estimate of these individual uncertainties, the confidence on visibility cannot be justified. Presumably the noise arises from a combination of Poisson counting statistics and Gaussian technical noise. A discussion of uncertainties and an estimate of their value, either in the caption of Fig2 or in the main text, should be included.

To clarify this concern : an extremely small uncertainty σ in these points would imply the outliers (e.g. G3=100nm; Counts=750) are meaningful (which they presumably are not) whereas an extremely large uncertainty would imply there is no information in these measurements. Taking a simplified version of the fit functon(which is alluded to rather than stated explicitly) $f(x) = S_0 + S_1 \cos(kx)$ with known k and x offset, for uniformly spaced measurements over complete periods then I believe this works out as $\sigma_{S_0} = \sigma/\sqrt{N}$ and $\sigma_{S_1}=\sigma\sqrt{2/N}$, assuming that the errors are uncorrelated and Gaussian distributed.

The term "Schrödinger cat" is used somewhat inconsistently, and is applied to both their 170 kDa nanoparticles delocalized over ~20 nm and to a 16 ug mechanical resonator (effective mass) delocalized over 10^-18 m. From this, I am unsure (for example) whether the authors would also call an atom delocalized over 0.5m (line 16) a "Schrödinger cat". The authors should define their usage of this term, being careful to capture how it applies to the Talbot-Lau geometry where the interpretation of "which way" is more subtle. Related to this, the term "complementary regimes" (line 27) is also not well-defined.

Near line 22, the mass unit ug is used, whereas elsewhere the authors use kDa, as is appropriate for their experiment. The vast difference in these units should be noted.

Mirror alignment (line 514): roll axis description appears geometrically inconsistent with beam configuration: even with context and careful reading, I have not been able to understand "mirror roll angles, around the molecular beam axis".

Thermal Stability: previous work e.g. OTIMA emphasised ~10nm thermal drift concerns of the mirrors due to e.g. UV absorption. Is this still relevant or is it now thoroughly mitigated?

Coherence Length (lines 59-64): Equation relating velocity spread to longitudinal coherence length is not explained for a general readership.

Collimation Description (line 68): "Collimating the beam to 100nm" is unclear and perhaps a typo, as presumably collimating is to some angular tolerance rather than spatial localisation.

Notes to authors:

1. Historical context
Add missing foundational reference Arndt et al. (1999) "C60 wave-particle duality" (doi:10.1038/44348) and, if appropriate, Chapman et al. (1995) Na2 molecules (doi:10.1103/PhysRevLett.74.4783) and Brezger et al. (2002) C70 (doi:10.1103/PhysRevLett.88.100404) to provide more complete historical timeline.

2. OTIMA development
Include key references describing your experimental platform: Haslinger et al. (2013) (doi:10.1038/nphys2542) and Dörre et al. (2014) (doi:10.1103/PhysRevLett.113.233001) to explain how the techniques evolved.

3. Statistical Analysis and Error Reporting
Add error bars to individual data points in Fig 2a or explain uncertainty sources (counting statistics and technical noise) in the figure caption. Discuss how visibility confidence intervals are determined.

4. Terminology Clarification
Define "Schrödinger cat state" usage consistently, distinguishing between different mass/separation regimes (170 kDa/~20 nm vs. 16 ug/10^-18 m) and explaining applicability to Talbot-Lau geometry where "which way" interpretation is more subtle.

5. Technical Clarifications
- Clarify mirror alignment description (line 514): "mirror roll angles, around the molecular beam axis" appears geometrically inconsistent
- Explain coherence length equation (lines 59-64) for general readership
- Correct collimation description (line 68): clarify whether "100nm" refers to angular or spatial constraint
- Note the vast difference between mass units (ug vs kDa) used for different experiments to help readers understand scale differences.

Version 1:

Reviewer comments:

Referee #1

(Remarks to the Author)
The revised manuscript reads very well. The authors have improved the didactial part of the paper and the supplement. It has gained in appeal to a general reader and the importance of the work is very clear also to a non specialist.
As far as I can see alsoe the concerns of the other referees have been met sufficiently in the revies version.
I support publication in its present form.

Referee #3

(Remarks to the Author)
I thank the authors for meticulously responding to my suggestions and the suggestions of the other referees. In my opinion, the manuscript is now ready for publication in Nature. This is a truly seminal result that should be celebrated, and it is fitting that the authors have achieved this in the International Year of Quantum Science and Technology. I give my congratulations and gratitude to the authors for their wonderful and inspiring work.

Referee #4

(Remarks to the Author)
I am confident that the authors have addressed my concerns, and those by other referees where we had overlap, and I therefore thoroughly recommend publication of this article.

Open Access This Peer Review File is licensed under a Creative Commons Attribution 4.0 International License, which permits use, sharing, adaptation, distribution and reproduction in any medium or format, as long as you give appropriate credit to the original author(s) and the source, provide a link to the Creative Commons license, and indicate if changes were made.
In cases where reviewers are anonymous, credit should be given to 'Anonymous Referee' and the source.
The images or other third party material in this Peer Review File are included in the article's Creative Commons license, unless indicated otherwise in a credit line to the material. If material is not included in the article's Creative Commons license and your intended use is not permitted by statutory regulation or exceeds the permitted use, you will need to obtain permission directly from the copyright holder.
To view a copy of this license, visit https://creativecommons.org/licenses/by/4.0/

Response to the Referee reports for manuscript 2025-06-15934

We would like to thank all reviewers for their positive feedback, and the appreciation of the importance of our work. Their valuable comments have helped us to improve the manuscript and in the following, we provide a point-by-point response to each of the reviewers' comments. We also indicate all corresponding changes in the revised manuscript.

In response to Reviewer 1: We thank the referee for their enthusiastic support and for emphasizing the importance of making our work accessible to a broad readership. In response, we have added a new explanatory paragraph in the Introduction that illustrates, in words, how a collapse model would predict our system to behave, and how our experiment rules it out. We then follow this example with the Introduction of the macroscopicity measure, as suggested.

. . . .

> **Reviewer 1:** "While the technical part of the paper is flawless and exhaustive, and the data and their analysis, including error analysis, are of very high quality, the general claim of the paper for a non-specialized audience should be strengthened and explained better and less technical and jargon-loaded."
>
> "The general claim roughly formulated is: we have tested the heart of quantum mechanics, the superposition principle, in a new regime and can exclude alternative theories a factor of 10 more sensitively than previous experiments. ... But what does that actually mean? What are the alternatives which are ruled out, and how does such an alternative model predict a degrading of interference visibility? I feel that an article in Nature should meet that challenge to make it understandable to at least a physics-educated reader what is really achieved."

Our reply: We thank the referee for their very positive assessment of our work and we are happy to make the paper more accessible to the broader audience. In response, we expanded the Introduction (lines 45–67) to explain how a collapse model predicts a reduction of the interference fringe visibility and how our experiment can rule this out. We have further revised the Discussion to include additional explanations intended for non-specialist readers (lines 229 - 251).

. . . .

> **Reviewer 1:** "Do something which one does normally in talks ... give handwaving explanations even if they are only a part of the truth. Tell a story like 'Theory A predicts...' or 'Theory B predicts...' ... Once such an example is formulated, one can explain what this particular theory would predict for the currently studied interference and which is the most critical experimental parameter (mass, size, time), and how the experiment falsifies that theory and why all other experiments cannot falsify that (possibly bizarre) theory."

Our reply: In the revised manuscript, the new paragraph (lines 45–67) provide the requested example and we emphasize why our experiments is essential in reaching to this parameter range. Also the discussion has been adapted (lines 229 - 251).

. . . .

> **Reviewer 1:** "In conclusion I strongly support publication of the present work in Nature after (didactical) changes. The topic is fascinating for a general audience, the experimental work is cutting etch and the claim is very well justified by the data – it should just be explained more didactically to a non-expert audience."

Our reply: We thank the referee for their strong support of our work. We have implemented the requested didactical changes and we believe that these revisions have improved the accessibility of the manuscript.

In response to Reviewer 2: We thank the referee for their careful reading and constructive feedback. In response to their comments, we have revised the Introduction and the Discussion to better highlight the key advances of this work and why it is qualitatively different from previous experiments. We have also expanded the historical context and updated numerous citations to our own previous work already in the abstract, to better position our current findings in the context of a wider research field.

. . . .

> **Reviewer 2:** "The experimental and computational implementations, data, and analysis look valid and appropriate, statistics seem correct and – including all analysis – are well described. The abstract is good. The discussion should point out what the central "punchline" finding of this work actually is that warrants publication in a very-high-impact journal"

Our reply: We thank the referee for their positive assessment and have taken the opportunity to highlight the key aspects in various places, some already mentioned here above. In response to this, we now also articulate explicitly the central 'punchline' in the revised Discussion, as asked by the referee. In brief, our work uses matter-wave interference to challenge Schrödinger's thought experiment with a previously inaccessible mass range and a new materials class: metal nanoparticles. This extends experimental tests of quantum mechanics to substantially heavier objects and importantly, this advance is *not* limited to sodium or to specially synthesized molecules. It establishes a completely new nanoparticle materials class for quantum experiments, providing a versatile, scalable platform that can accelerate progress toward still higher masses and greater structural complexity. Enabled by a new interferometer (cw-UV beam splitting, high-mass–sensitive detection, and metal-cluster beam generation), the platform delivers an order-of-magnitude increase in quantum macroscopicity and reaches de Broglie wavelengths compatible with those of biological structures, already overlapping with large proteins and even slow viruses. While additional development will be needed for genuine

biological nanostructures, our results indicate no fundamental stop sign imposed by mass alone, or by short de Broglie wavelengths, clarifying the qualitative significance beyond the technical implementation.

. . . .

> **Reviewer 2:** "Own previous work is not very well summarized nor cited, corresponding updates to the introduction and experimental details would be useful."

Our reply: We thank the referee for pointing this out. We had initially kept the number of self-citations intentionally low, to leave room to refer to work of others. In the revised manuscript, we have now added the most relevant references already in the abstract and added additional comments in the Introduction to provide a historical context.

. . . .

> **Reviewer 2:** "Overall, to my mind, this manuscript can almost be published as is, i.e., with updates as stated above. However, to me this is, albeit surely an heroic effort, a somewhat "incremental" improvement over previous work by the same authors. Unless I am overseeing a truly significant qualitative improvement over previous work (see above), I suggest to publish this work in a more specialized journal."

Our reply: Again, we thank the referee for this positive feedback. We have implemented the suggested updates. As outlined in the response to the other reviewers, this work shows indeed a new experimental platform, addressing an entire material class that has never been used by anyone in quantum interference experiments before. Our experiment is operating in a parameter regime that boosts the state of the art by an order of magnitude in de Broglie wavelength and macroscopicity, with the potential to be scalable, as demonstrated in lines 283-285, Fig. 3 and also in the Suppl. Info.

In response to Reviewer 3: We thank the referee for their very positive and encouraging evaluation. Following their suggestions, we have broadened our discussion of collapse models and clarified the methodology in the main text and the supplement.

. . . .

> **Reviewer 3:** "I feel that it is important for the authors to broaden their discussion of the constraints their results place on proposed modifications to quantum mechanics, and how these constraints compare to those from other experiments."
> "The discussion currently focuses on macroscopicity, which is useful but limited. ... Since CSL is defined by two independent parameters (rate and correlation length), two experiments with the same macroscopicity may probe different regions of this space. ... I therefore suggest including an exclusion plot for the CSL parameter space, as in Ref. [5]."

Our reply: We consider the macroscopicity value to be the best suited quantity to place our experiment in a broad, model-independent context for generic modifications of quantum mechanics. At the same time, we agree that this metric does not capture the full parameter space of specific models such as CSL. We now explicitly discuss the excluded parameter space and provide an exclusion plot in the Supplementary Information. Since CSL is a model whose relevant parameters have already been largely excluded by non-interferometric tests, at least for a broad class of underlying noise models, we chose to keep the main text focused on macroscopicity, while addressing CSL and other models in the supplement, see also our responses to the subsequent comments. We have also added a discussion (line 45-56) and a citation of the 2003 review on CSL models by Bassi and Ghirardi to the Introduction.

. . . .

> **Reviewer 3:** "The authors state in the abstract that their record macroscopicity value provides 'the most stringent exclusion limit for generic macrorealistic modifications of the Schrödinger equation to date.' ... I believe this statement requires further qualification. It is possible that another experiment with lower macroscopicity could rule out a larger area of CSL parameter space. I suggest that the authors check this or clarify the statement in the abstract."

Our reply: While our statement referred to the generic, model-independent assessment of macrorealistic modifications in terms of the macroscopicity, it is of course true that each concrete model comes with its own parameter space. To avoid any misinterpretation of our statement we removed the mentioned clause from the abstract.

. . . .

> **Reviewer 3:** "In addition to decohering quantum superpositions, many proposed modifications to quantum mechanics (e.g., CSL) also would lead to spontaneous heating. Experiments that bound this spontaneous heating therefore also constrain the CSL parameter space. ... I believe that the authors should compare the constraints from their work to constraints from experiments that bound spontaneous heating. A relevant reference is Vinante et al., Phys. Rev. Lett. 125, 100404 (2020). ..."

Our reply: We are well aware that non-interferometric tests of the CSL model (searches for anomalous heating or spontaneously emitted radiation) exclude relevant regions of the CSL parameter space, and that the basic form of the model can therefore be considered largely ruled out as a solution to the measurement problem. However, as noted by the reviewer, matter-wave interference represents a genuine quantum test. Genuine quantum tests are essential as they probe what the inventors of CSL models wanted to probe - the possible breakdown of the superposition principle. Moreover, quantum tests are independent of any assumption about 'noise' and 'thermalization' in modified CSL models, which might limit the observability of CSL effects in non-interferometric tests. As CSL is only one among many possible modifications of quantum mechanics, our main text emphasizes the generic and model-independent macroscopicity rather than

focusing on CSL. However, we agree with the referee that it is valuable to point out this complementarity, and we now include a discussion of spontaneous-heating constraints in the Supplementary Information together with an updated exclusion plot from molecule interferometry for the CSL parameter space.

....

> **Reviewer 3:** "Along with CSL, it would be very valuable if the authors could comment on the constraints put by their experiment on other specific models. Relevant examples include the Kafri–Taylor–Milburn model, Oppenheim's postquantum theory of classical gravity, and the entropic model of gravity in Carney et al."

Our reply: Indeed, a number of gravitationally inspired collapse models have been proposed. Arguably the most prominent one is the Diósi model (or Diósi–Penrose model), which is based on an assumed gravitational self-interaction of mass in a superposition state. The corresponding master equation has the same structure as that of CSL. To avoid unphysical heating rates it requires smearing the mass distribution of the particle by a scale parameter R_0 many orders of magnitude greater than the actual size of the nuclear mass distribution. As discussed in the following reply, any reasonable value of R_0 is much greater than the lower bound that can be provided by matter wave interferometry.

The mentioned Kafri-Taylor-Milburn model treats gravity as a classical measurement-and-feedback channel. In a linearized approximation it yields position decoherence that is "consistent with Diósi's model" [New. J. Phys. 16, 065020 (2014)]. It can thus be viewed as in independent motivation for the Diosi master equation, but it makes no different predictions, nor does it provide estimates for the strength of the effect.

Oppenheim's postquantum theory of classical gravity develops a general framework for hybrid classical-quantum dynamics, with the aim of keeping the gravitational space-time dynamics classical when coupling it consistently to quantum particles acting as mass sources. He shows that any consistent hybrid dynamics is characterized by a trade-off between decoherence on the quantum side and diffusion on the classical system. In the nonrelativistic limit, Oppenheim's framework can be brought into the general form of the CSL master equation with an unspecified decoherence kernel. Assuming Netwonian scaling $\propto 1/|x - y|$ one again ends up with the form of the Diosi master equation.

The entropic model of gravity by Carney et al. models gravity as an entropic force produced by mediator qubits, located at a lattice with constant a, whose energies are determined by the particle positions. Also this model gives rise to position decoherence accompanied by anomalous heating. The characteristic decoherence rate (66) in [Phys. Rev. X 15, 031038 (2025)] has again the same form (up to a factor of order unity) as the Diósi–Penrose rate, except that the smearing scale R_0 is replaced by the lattice constant a.

The mentioned models can thus all be seen as alternative justifications or substantiations of the form of the Diósi master equation. But they do not make independent predictions or provide independent motivations for the strength of the effect. These models are therefore constrained at the same level as the Diósi model, discussed in our next reply.

....

> **Reviewer 3:** "Is the present experiment able to test the Diosi–Penrose model, or are there prospects for doing this with future upgrades?"

Our reply: The present experiment cannot probe the Diósi–Penrose model, nor does any other quantum interference experiment so far. The maximum gravitational decoherence rate is $\Gamma_{\max} \simeq 6GM^2/(\hbar R_0)$, with G the gravitational constant, M the particle mass, and R_0 the smearing scale parameter mentioned above. Given our interference time of about 12 ms, we would be sensitive to this gravitational decoherence only for $R_0 < 10^{-21}$ m, much smaller than the natural scale, the size of an atomic nucleus. At the same time, non-interferometric measurements show that the model is empirically consistent only with R_0 being more than ten orders of magnitude greater [Donadi et al., Nat. Phys. 17, 74 (2021)]. To become sensitive to this latter scale, one would have to demonstrate the spatial delocalization of a 10^8 Da particle over ten seconds - or more massive for shorter time scales. This is challenging and will indeed require a different experiment. However, the versatility of our particle source and the diffraction elements are still compatible with such tests. We now cite Diosi's seminal work in the Introduction and discuss the prospects of observing gravitational decoherence in the supplement.

. . . .

> **Reviewer 3:** "The introduction might benefit from a brief explanation of the motivation for collapse models from the point of view of the measurement problem. Reference [15] has a particularly good discussion of this point that could be cited here."

Our reply: We now mention the measurement problem as a motivation for collapse models in the Introduction (lines 45 -49). Our main motivation is to test the quantum superposition principle in a model-independent way, rather than focusing on a specific model such as CSL. Reference [Bassi. et al., Rev. Mod. Phys. 85, 471(2013)] (formerly reference [15]) provides an excellent review of collapse models and the measurement problem, and we already cite it prominently in the Introduction. In addition, and in line with Reviewer 1's request, we have added an explanatory paragraph (lines 45–67) that illustrates a specific collapse scenario in plain words, to provide non-specialist readers with a concrete example without recreating the full discussion of the measurement problem.

. . . .

> **Reviewer 3:** "In Figure 2(b), both the classical and quantum theory curves are scaled by the same global factor of 0.78 . . . I would tend to think that such factors might have a different effect on the contrast for the classical vs. quantum case. If I am mistaken, can the authors please add a brief explanation as to why they should be the same?"

Our reply: We use the same global scaling factor because the effects of interferometer misalignment, gravitational and rotational phase averaging, mechanical vibrations, and

scattering of gas particles and thermal radiation indeed all act in the same way in the quantum and the classical description (line 184 - 188). This can be seen by representing the dynamics in phase space by means of the Wigner function and the phase-space distribution, respectively. Each of these effects then amounts to a blurring in phase space, defined by the same integral kernel in the quantum and the classical formulation, see [Hornberger et al., Phys. Rev. A 70, 053608 (2004)] and [Stibor et al., Laser Phys. 15, 10 (2005)]. This would no longer be valid if the particle's motion became entangled with internal degrees of freedom due to environmental interactions, which is however not the case for the processes occurring in our experiment.

That said, we emphasize, that the scaling is applied only in Fig 2(b), and only to ease visual comparison of the data with the quantum vs. the classical model. The quantitative macroscopicity assessment is done with the *unscaled* measurement data.

. . . .

> **Reviewer 3:** "In Supplement Figure 5, the description of the curves in the caption does not seem to match the legend on the plot."

Our reply: We thank the referee for spotting this inconsistency. We have corrected the caption of Supplementary Fig. 5 to match the legend and ensure consistency between description and plot.

. . . .

> **Reviewer 3:** "The authors use an adaptive Gaussian low-pass filter to denoise the time-of-flight data. Could the authors say more about how the 'adaptive' part of the filtering algorithm works? Have the authors verified that their end results do not significantly depend on small changes in the parameters of the filter?"

Our reply: The Gaussian low pass filter is adaptive in the sense that its width is iteratively increased until the error of the smoothed data is below a predefined threshold. The smoothed data are only used to find the initial conditions for fitting the velocity distribution and for visualization purposes in Suppl. Fig. 2. The fit used for the velocity evaluation is directly applied to the raw data and because of this the results do not depend on the smoothing parameters. We have adapted the description of our velocity analysis in the revised manuscript to more accurately reflect this.

. . . .

> **Reviewer 3:** "The manuscript is well-written . . . I believe that it would make an outstanding contribution to Nature, provided that the detailed comments below are addressed."

Our reply: We thank the referee for this very positive assessment. We have carefully addressed all of the detailed comments raised, as outlined in our responses.

In response to Reviewer 4: We thank the referee for their very positive overall assessment, highlighting both the quality and groundbreaking nature of the experiment. We are also grateful for the careful and detailed suggestions, which have helped us to further improve the clarity and accessibility of the manuscript. In the revised version, we have expanded the historical context, clarified the discussion of technical aspects as outlined in the individual responses, and refined our terminology regarding "Schrödinger cat states."

. . . .

> **Reviewer 4:** "This work merits publication in Nature following minor revision to address the issues outlined below. The work presented is of extremely high quality and the experiment truly groundbreaking."

Our reply: We thank the referee for this very positive assessment.

. . . .

> **Reviewer 4:** "The discussion of previous work is a little sparse and may not give sufficient context for this new result. . . . The most notable absence is the seminal 1999 paper Arndt et al. 'C60 wave-particle duality' . . . Making more clear the timeline of this work will also make their claims about what they may achieve in the future more believable."

Our reply: In our original submission, we deliberately limited self-citations to give room to other groups and authors. We agree, however, that certain contributions are important and we have added them, to give readers a clearer view of the timeline of progress, while trying to keep the ratio of self-citations low.

. . . .

> **Reviewer 4:** "Additionally, the manuscript appears to under-cite the development of their experimental platform. The experiment appears to use the OTIMA interferometer, yet the manuscript omits key references describing its design and capabilities . . . While the current application to metal nanoparticles is novel, readers would benefit from understanding how the experimental techniques evolved and were validated in previous studies using the same apparatus."

Our reply: We would like to clarify that the present experiment is *not* based on the OTIMA platform. While both OTIMA and MUSCLE make use of UV light, the two are

distinct interferometers with different capabilities and techniques in different laboratories and using different experimental ideas. OTIMA was an interferometer with pulsed gratings in the deep ultraviolet (10 ns, 157 nm), exploiting the stability of a short stable single mirror to demonstrating the wave-particle duality for organic molecules prepared in supersonic beams. This idea was not simply scalable to the same high masses that we achieve in MUSCLE, mostly because of the lack of particle suitable sources.

This is why MUSCLE was built: it employs continuous gratings at a much more readily accessible wavelength of 266 nm, and the gratings are separated by a Talbot distance of 1 m, using individually aligned mirrors, requiring sophisticated vibration isolation and alignment techniques. The interferometer is not only more than 10 times larger than OTIMA, but also compatible with a much broader range of continuous sources of larger metal clusters. In contrast to OTIMA, MUSCLE can accept velocity spreads more than 10 times wider, a feat without which the experiments would not have worked. This is due to operating in real space (MUSCLE) rather than in the time domain (OTIMA). The price to pay is a more demanding compensation scheme for gravity and vibrations in MUSCLE. MUSCLE, shares grating ideas with OTIMA (UV photo-depletion), other experimental concepts with LUMI (long baseline, continuous gratings), but adds to both new tools such as laser technology, clusters sources, mass selection in a regime where commercial detectors are severely limited. Thus, although we have benefited from the integrated experience with all earlier matter-wave experiments in the group, the current work is clearly an independent platform.

. . . .

> **Reviewer 4:** "On a more technical note, the authors claim a visibility and provide an uncertainty on this estimate. However, the confidence in this visibility estimate depends critically on the individual uncertainties in counts . . . Without an estimate of these individual uncertainties, the confidence on visibility cannot be justified. . . . A discussion of uncertainties and an estimate of their value, either in the caption of Fig. 2 or in the main text, should be included."

Our reply: We thank the referee for the careful comment on our error statistics of Fig 2. Our original analysis already tied the reported visibility to the individual per-scan uncertainties from the nonlinear least square sine fits, each based on maximum likelihood estimation. The latter also yield 1σ confidence intervals by assuming that the counts are independent random events with finite variance (as in case of Poissonian or Gaussian distributions). However, a re-inspection, triggered by the referee's note, revealed a minor normalization issue in the bin-wise combination of individual scans that overestimated the errors in the power scan (Fig. 2(b)). We have corrected this and now state the parameter estimation procedure with weighted least squares explicitly.

For each power bin we compute the inverse-variance weighted mean of the individual visibilities v_i with per-point 1σ bounds $(v_{i,\mathrm{lb}}, v_{i,\mathrm{ub}})$ as

$$\mu = \frac{\sum_i w_i v_i}{\sum_i w_i}. \tag{1}$$

where

$$\sigma_{i,-} = v_i - v_{i,\mathrm{lb}}, \quad \sigma_{i,+} = v_{i,\mathrm{ub}} - v_i, \quad \sigma_i = \tfrac{1}{2}(\sigma_{i,-} + \sigma_{i,+}), \quad w_i = \frac{1}{\sigma_i^2}, \tag{2}$$

The uncertainty of the mean is

$$\sigma_\mu = \frac{1}{\sqrt{\sum_i w_i}}, \tag{3}$$

and, to reflect mild asymmetry, we also report side-specific bars

$$\sigma_{\mu,-} = \frac{1}{\sqrt{\sum_i \sigma_{i,-}^{-2}}}, \qquad \sigma_{\mu,+} = \frac{1}{\sqrt{\sum_i \sigma_{i,+}^{-2}}}. \tag{4}$$

As a consistency check, we evaluate the reduced chi-square,

$$\chi^2_{\text{red}} = \frac{1}{N-1} \sum_i \frac{(v_i - \mu)^2}{\sigma_i^2}, \tag{5}$$

and apply a Birge-type inflation only if $\chi^2_{\text{red}} > 1.5$ (we never shrink for $\chi^2_{\text{red}} < 1$).

This correction does not affect the scientific conclusions at all. We also like to emphasize, that Fig. 2 is purely illustrative and was *not* used in any downstream calculations (e.g. macroscopicity), which rely on the raw data (3,895 points) as described in the Supplementary Information. Fig. 2 and its caption have been updated accordingly, and the Methods now describe in detail how the plotted points and uncertainties are obtained.

. . . .

> **Reviewer 4:** "The term 'Schrödinger cat' is used somewhat inconsistently ... The authors should define their usage of this term, being careful to capture how it applies to the Talbot–Lau geometry where the interpretation of 'which way' is more subtle. Related to this, the term 'complementary regimes' ... is also not well defined."

Our reply: We agree that the term "Schrödinger cat" has been used differently across literature. Here, we use the term in a broad, qualitative sense, referring to genuine quantum state in which a massive and complex object - with many atoms and internal degrees of freedom - is prepared in a quantum superposition of classically distinct states. This definition encompasses both nanoparticle interferometry and macroscopic resonator experiments, even though the relevant parameters (mass, delocalization distance, coherence time) differ vastly across the platforms. This is mentioned both in the Introduction and in the Discussion. For clarity, we have removed the term from the title and added a sentence in line 37-40 that explains our definition in a concise way.

. . . .

> **Reviewer 4:** "Near line 22, the mass unit μg is used, whereas elsewhere the authors use kDa ... The vast difference in these units should be noted."

Our reply: We have added the corresponding value in Da after the μg reference (line 29).

. . . .

> **Reviewer 4:** “Mirror alignment (line 514): roll axis description appears geometrically inconsistent with beam configuration ... I have not been able to understand ‘mirror roll angles, around the molecular beam axis’.”

Our reply: We have clarified the description of the roll angle in the revised manuscript. The “roll angle” refers to a rotation of the mirror surface around an axis parallel to the incident molecular beam, which would tilt the grating with respect to gravity. For further clarity, we have added the coordinate system with all degrees of freedom to SI Fig. 1b.

. . . .

> **Reviewer 4:** “Thermal Stability: previous work e.g. OTIMA emphasised 10 nm thermal drift concerns ... Is this still relevant or is it now thoroughly mitigated?”

Our reply: In the present experiment, we reflect continuous-wave 266 nm light from three separate optics to form the standing light waves. Their transverse positions are actively monitored and stabilized on a nanometer scale using piezo actuators (SmarAct). Residual drifts have not posed additional problems during the measurement time, which is owed to outstanding vibration isolation in ultra-high vacuum.

. . . .

> **Reviewer 4:** “Coherence Length (lines 59–64): Equation relating velocity spread to longitudinal coherence length is not explained for a general readership.”

Our reply: We thank the referee for pointing this out. Since the longitudinal coherence length is not used further in the manuscript and the original sentence (line 88-90) was rather technical, we have removed it in the revised version.

. . . .

> **Reviewer 4:** “Collimation Description (line 68): "Collimating the beam to 100 nm" is unclear and perhaps a typo, as presumably collimating is to some angular tolerance rather than spatial localisation.”

Our reply: We thank the referee for spotting this. The original wording was misleading. What we control is the angular collimation of the nanoparticle beam, not a spatial confinement to 100 nm. We have corrected this in the revised manuscript (line 90-93).

Response to the Referee reports for manuscript 2025-06-15934A

On behalf of all co-authors, we would like to express our sincere thanks for your careful reading of our manuscript and for the very constructive comments provided in the previous round. We are especially grateful for your positive assessment of the scientific significance of the work and for your statement that, with the implemented changes, the manuscript is ready for publication in Nature. Your suggestions substantially improved the clarity, completeness, and balance of the paper. We very much appreciate the time and expertise you invested in this review process.
